# Bacterial community structure of early-stage biofilms is dictated by temporal succession rather than substrate types in the southern coastal seawater of India

**DOI:** 10.1371/journal.pone.0257961

**Published:** 2021-09-27

**Authors:** T. J. Sushmitha, Meora Rajeev, P. Sriyutha Murthy, S. Ganesh, Subba Rao Toleti, Shunmugiah Karutha Pandian

**Affiliations:** 1 Department of Biotechnology, Alagappa University, Karaikudi, Tamil Nadu, India; 2 Water and Steam Chemistry Division, Bhabha Atomic Research Centre Facilities, Kalpakkam, Tamil Nadu, India; 3 Department of Chemistry, Scott Christian College, Nagercoil, Tamil Nadu, India; National Cheng Kung University, TAIWAN

## Abstract

Bacterial communities colonized on submerged substrata are recognized as a key factor in the formation of complex biofouling phenomenon in the marine environment. Despite massive maritime activities and a large industrial sector in the nearshore of the Laccadive Sea, studies describing pioneer bacterial colonizers and community succession during the early-stage biofilm are scarce. We investigated the biofilm-forming bacterial community succession on three substrata viz. stainless steel, high-density polyethylene, and titanium over 15 days of immersion in the seawater intake area of a power plant, located in the southern coastal region of India. The bacterial community composition of biofilms and peripheral seawater were analyzed by Illumina MiSeq sequenced 16S rRNA gene amplicons. The obtained metataxonomic results indicated a profound influence of temporal succession over substrate type on the early-stage biofilm-forming microbiota. Bacterial communities showed vivid temporal dynamics that involved variations in abundant bacterial groups. The proportion of dominant phyla viz. *Proteobacteria* decreased over biofilm succession days, while *Bacteroidetes* increased, suggesting their role as initial and late colonizers, respectively. A rapid fluctuation in the proportion of two bacterial orders viz. *Alteromonadales* and *Vibrionales* were observed throughout the successional stages. LEfSe analysis identified specific bacterial groups at all stages of biofilm development, whereas no substrata type-specific groups were observed. Furthermore, the results of PCoA and UPGMA hierarchical clustering demonstrated that the biofilm-forming community varied considerably from the planktonic community. Phylum *Proteobacteria* preponderated the biofilm-forming community, while the *Bacteroidetes*, *Cyanobacteria*, and *Actinobacteria* dominated the planktonic community. Overall, our results refute the common assumption that substrate material has a decisive impact on biofilm formation; rather, it portrayed that the temporal succession overshadowed the influence of the substrate material. Our findings provide a scientific understanding of the factors shaping initial biofilm development in the marine environment and will help in designing efficient site-specific anti-biofouling strategies.

## Introduction

Marine biofilms–an undesirable accumulation of microorganisms provide a captivating environment to analyze and record the complex bacterial interactions. Once immersed in the marine environment, surfaces of any material quickly adsorb organic molecules and form a conditioning film [1, 2]. This nutrient-rich organic layer offers an ideal environment for the colonization of microorganisms viz., bacteria, algae, diatoms, archaea, and protozoa that get embedded in a self-produced matrix of extracellular polymeric substances (EPS) interspersed with fluid channels [3–5]. This complex and dynamic community is known as biofilm and is often referred to as microfouling in the marine environment. The architecture of the biofilm grants a favourable mode of existence to microorganisms and presents a viable strategy to inhabit new niches [6]. Marine biofilms are also acknowledged for their significant role in the productivity and functioning of coastal ecosystems as they contribute to the fundamental ecological processes including nitrogen cycling and degradation of organic matters [7, 8]. On the other hand, marine biofilms also play an antagonistic role by inducing other complex phenomena such as biofouling and biocorrosion [9, 10] that lead to imposing several deleterious consequences in maritime industries, especially cooling systems of nuclear power plants.

Coastal positioned cooling systems utilize a large volume of seawater as a key source for waste heat removal [11, 12]. The traversing seawater through power plants generally contains a wide spectrum of microorganisms that tend to settle and form biofilms on the surface of the condenser and other operational machinery [13]. This unwanted biological growth causes several detrimental consequences including mechanical blockage of condensers, reduction in the flow rate of pipelines, impairment in heat transfer efficiency of heat exchangers and premature replacement of structural components [14]. The economic impact of microfouling has been widely examined [9]. It is estimated that the economic losses from corrosion are about USD 2.5 trillion per year, approximately 3% of gross domestic product [15]. Similarly, Walker et al. reported that the related cost associated with the condenser fouling is in the order of USD 0.4 to 4.4 million [16]. Overall, marine biofilms are considered fascinating ecosystems from both ecological and economical perspectives, and therefore identifying the pioneer colonizers, and understanding the key factors influencing the community composition, development, and succession in their early stage will be a worthwhile contribution.

In recent decade, there has been a surge in studies on biofilm ecology and functions, with descriptions of their diversity and community composition [e.g. 4, 8, 17–23]. Based on their analyses on biofilm-forming microbiota, the structure of marine biofilms is not a mere reflection of planktonic bacteria present in the surrounding seawater whereas; it is influenced by several factors including location, prevailing environmental factors, and substrate (type, orientation, and colour). Some of the previous studies have shown that the fundamental mechanisms that control initial bacterial assembly are governed by species sorting (especially the effect of different substrata) whereas, others have shown that this phenomenon is neutral [24]. For example, Meier et al. investigated the effect of engineering surfaces at Cayman Trough and concluded that substrate types harboured varied bacterial communities [25]. Similarly, another study inferred that the effect of species sorting on substrate type was more prominent during the early-stage biofilm development at the Red Sea cold seep system [26]. In contrast to these studies, Bellou et al. and Lee et al. observed that depth and *in situ* environment, respectively play a major role than substrate type in determining the biofilm-forming microbiota [27, 28].

Identification of typical dominant colonizers, on the other hand, is another major concern. Intriguingly, a large inconsistency has been ascertained about the dominant bacterial taxa during the early biofilm development by the studies conducted in various marine environments. For instance, in few studies [17, 23, 29–31] genus *Roseobacter* (*Alphaproteobacteria* class) was observed as typical dominant colonizers, while few other studies [8, 19, 26, 30, 32] observed genus *Alteromonas* (*Gammaproteobacteria* class). This part is particularly interesting because, besides the species sorting and neutral effects, primary colonizers decide the recruitment of secondary and tertiary colonizers.

So far, studies have been conducted either to decipher the influence of substrate types and environmental factors on biofilm-forming community [25–28] or to analyze the temporal succession pattern of biofilm-forming microbiota assembled on the same substrate material [8, 23, 33]. Integrated studies examining the temporal succession pattern of the early-stage biofilm-forming bacterial community on various artificial substrata are rather limited, particularly in a peninsular country like India, where biofouling is a major concern. Furthermore, the majority of earlier studies in the Indian context have been conducted to determine macrofouling communities using classical methods [34–36]. However, studies investigating the succession pattern during the early stages of biofilm development on various artificial substrata using modern sequencing technologies are not yet carried out.

To fill this lacuna, we investigated the bacterial community composition of the early-stage marine biofilms formed on three substrate types for initial 15 days in a coastal environment, located in the Laccadive Sea, Indian Ocean. The main objectives we aimed in this study were to: (i) catalogue the primary colonizers responsible for causing microfouling in the southern coastal region of India, (ii) understand the impact of substrate types and successional stages on the early-stage biofilm-forming bacterial community composition and structure, and (iii) compare the biofilm-forming and planktonic bacterial community. The outcomes of this study will enlighten the scientific community to understand the interactions among marine microbes during the initial stage of biofilm development.

## Materials and methods

### Ethical statement

The Nuclear Power Corporation of India (NPCIL) and the authorities of Kudankulam Nuclear Power Plant granted all the necessary permissions to conduct sampling and research work. No Animal Care and Use Committee approval was necessary for this study as no vertebrate sampling was carried out.

### Study site and experimental setup

Field sampling was conducted in the intake area of a nuclear power plant (08°10′08′′N, 77°42′45′′E) located in the coastal region of Kudankulam, Tamil Nadu, India (**S1a Fig in S1 File**). A detailed description of the studied power plant is provided in our previous study [12]. The study site is located in the distal end of the Gulf of Mannar Biosphere Reserve and is endowed with rich marine diversity with various species of microbes, corals, fishes, crustaceans, mollusks, and sponges [37, 38].

To understand the bacterial community dynamics during early-stage marine biofilm development, succession pattern was investigated consecutively for 15 days (during October 15–30, 2020) on three different substrata viz., stainless steel (SS, 316L), high-density polyethylene (HP), and titanium (Ti, Grade1). The selection of substrate materials used in our study was based on their: (i) common applications in maritime industries (e.g. SS and Ti used as condenser coating material in power plants and HP for designing pipelines) and (ii) varied surface characteristics. Virgin coupons (6 each) of these substrata with a size of 15 cm × 10 cm × 0.2 cm (total surface area ~ 300 cm^2^) were embedded in a custom-made polypropylene frame. Prior to fixing on the polypropylene frame, all coupons were rinsed with 70% ethanol and were gently sandblasted to facilitate microbial adhesion. Each frame was designed in a way that it holds 18 coupons (6 coupons × 3 substrata) (**S1b Fig in S1 File**). A total of 12 frames were immersed vertically in the seawater at a depth of 1 m using PVC jacketed steel ropes. Two frames (technical replicates) were retrieved on each of the six time points: 1, 3, 6, 9, 12 and 15 days. Thus, at every sampling occasion, a total of 12 coupons were analyzed for each of the investigated substrate type. Besides the three substrata, acrylic coupons with size of 5 cm × 2 cm × 0.2 cm were also embedded onto the polypropylene frames for microscopic analysis (**S1b Fig in S1 File**). After retrieval, the acrylic coupons were fixed on-site using 4% formaldehyde (v/v) and were brought to the laboratory under ice-cold condition. In addition to marine biofilms, surface seawater (SW) from the close periphery of the immersed frames was also collected on all sampling occasions to record the planktonic fraction.

### Environmental parameters and seawater nutrients

Delicate environmental parameters such as pH, temperature, and salinity were measured *in situ* with a portable pH meter (Eutech Instruments, Singapore), standard mercury thermometer, and salinity refractometer (Atago Pvt. Ltd., India), respectively. For dissolved oxygen (DO), seawater was collected in 300 mL BOD (biochemical oxygen demand) bottles, and DO content was fixed on-site using 1 mL of both Winkler-A and Winkler-B reagents. The fixed DO was measured using the modified Winkler titration method as described by Carpenter (1965) [39]. Concentrations of seawater nutrients–soluble reactive phosphorous (PO_4_^-^), nitrite (NO_2_^-^), and nitrate (NO_3_^-^) were measured as previously suggested [40, 41].

### Microscopic analysis

The development of the microbial growth on acrylic coupons was visualized using field-emission scanning electron microscope (FESEM, SUPRA 55VP; Carl Zeiss, Germany) as described in our previous study [12]. Briefly, the bacterial biomass fixed on the acrylic coupons was dehydrated by passing through the graded ethanol concentrations (20, 40, 60, 80, and 100%; 10 min each). The samples were then gold-sputtered and observed under the microscope.

### Nucleic acid extraction, PCR amplification, and Illumina sequencing

Immediately after the retrieval of coupons, the loosely attached fraction of biofilms was removed by carefully rinsing with sterile seawater (0.22 μm filtered and autoclaved). Bacterial biomass assembled on the studied substrata was then scraped using sterile toothbrushes and was resuspended in sterile seawater. The bacterial cells of representative biofilm (12 coupons/substrata/sampling occasion) and seawater samples were concentrated onto 0.22 μm nitrocellulose filters (Millipore, Massachusetts, US) and frozen at −80°C until further processing. The DNA was extracted using PowerWater® DNA Isolation Kit (MoBio Laboratories Inc., USA) following the manufacturer’s instructions. The isolated DNA was eluted in an aliquot of 50 μL PCR-grade water and the quality and quantity were measured by picogreen (Invitrogen) using Victor 3 fluorometry. The extracted metagenomic DNA was used as a template for the preparation of 16S rRNA gene amplicon libraries following standard Illumina library preparation procedure as described in our earlier studies [42, 43]. Briefly, the V3-V4 hypervariable regions of the 16S rRNA gene were initially amplified using a universal pair of primer: 341F (5ʹ-CCTACGGGNGGCWGCAG-3ʹ) and 805R (5ʹ-GACTACHVGGGTATCTAATCC-3ʹ) resulting in an amplicon length of ~464 bp [44]. A second PCR was then carried out to attach dual Illumina barcode indices and adapters using the Nextera XT library preparation kit (Illumina, San Diego, CA). The final library size was validated using a DNA 1000 chip on Bioanalyzer 2100 (Agilent technologies, California, US) and was quantified using dsDNA binding fluorescent dyes (Qubit dsDNA HS Assay Kits, Life Technologies, USA). The quantified libraries were then sequenced through 300×2 bp paired-end sequencing using a v3 reagent kit (Illumina, San Diego, CA, USA) on an Illumina MiSeq platform at Macrogen Inc. (Seoul, Korea).

### Bioinformatics and biostatistics analyses

The demultiplexed data obtained from the service end were loaded in the Quantitative Insights Into Microbial Ecology (qiime2 v. 2020.11) [45] environment as a manifest file using the qiime tools import–type “SampleData [Paired End Sequences with Quality]” and–source-format PairedEndFastqManifestPhred33V2. The sequences were then denoised using DADA2 [46], aligned with MAFFT [47], and the phylogenetic tree was built with default parameters using fasttree2 [48]. The obtained features were taxonomically assigned with the q2-feature-classifier using the Greengenes 13_8 reference sequences (Greengenes 13_8: 99_OTUs) [49]. Adequate sampling depth was assured by constructing rarefaction curves and the core-diversity metrics (both alpha and beta) were calculated (qiime diversity core-metrics-phylogenetic) using the rarefied feature table.

Prior to all analyses on bacterial community data, Shapiro-wilk test was performed to determine the normality distribution of the data. The differences in alpha-diversity indices were calculated using Kruskal-Wallis test. The variability in biofilm and seawater microbiota was examined using principal coordinate analysis (PCoA) performed with both weighted and unweighted UniFrac distance measures in qiime2. The multivariate differences in the bacterial community composition among succession days, substrate type and biofilm-forming and seawater microbiota were analyzed using permutational multivariate analysis of variance (PERMANOVA) followed by a pairwise test. Both Kruskal-Wallis test and PERMANOVA were conducted in qiime2. One-way ANOVA to ascertain significant differences for taxon abundance and the UPGMA tree with 9999 permutation were produced in PAST software. Spearman’s rank correlations among bacterial phyla were calculated in R version 3.6.2. All figures were generated using the plugin ggplot2 in R.

Further, linear discriminant analysis (LDA) effect size (LEfSe) [50] was used to identify the discriminative features that varied significantly among succession days, substrate types, and between biofilm-forming and seawater microbiota. The analysis was conducted at the online Galaxy interface (http://huttenhower.sph.harvard.edu/galaxy), wherein default parameters of Kruskal-Wallis (0.05) and LDA score of greater than 3 was used to identify the discriminative features.

### Deposition of raw NGS reads in public repository

The Illumina-generated paired-end raw reads (.fastq files) for this study are available in the NCBI database under the accession codes: SRR14741817- SRR14741840 (Short Read Archive; SRA) and PRJNA735217 (BioProject).

## Results

### Environmental parameters

The physico-chemical characteristics and nutrients content of seawater collected throughout the sampling occasions are summarized in **Table 1**. During all the sampling occasions, the temperature of the seawater was found to be in the range of 26°C to 27.5°C and the pH ranged between 8.01 and 8.15. Content of DO was detected in the range of 5.8 to 6.9 mg L^-1^. Similarly, concentration of inorganic nutrients ranged from 0.33 to 1.44 μM for nitrite, 4.99–15.22 μM for nitrate, and 0.62–2.15 μM for soluble reactive phosphorous. No significant differences among the environmental parameters and nutrients content were observed during the sampling occasions. Regarding the development of biofilm biomass, there was an average increase in 7.6 ± 3.7 g per bare plate on SS, 10.2 ± 4.5 g on HP, and 5.4 ± 2.1 g on Ti.

**Table 1:** Environmental parameters and nutrients content of seawater.

Parameters	Day 1	Day 3	Day 6	Day 9	Day 12	Day 15
Temperature (°C)	26	27.5	27.5	26.5	27	27.5
pH	8.13	8.09	8.01	8.15	8.09	8.08
Salinity (g kg ^-1^)	35	35	35	35	35	35
DO (mg L ^-1^)	5.80 ± 0.22	6.11 ± 0.22	6.74 ± 0.22	6.89 ± 0.44	6.42 ± 0.22	6.27 ± 0.44
Nitrite (μM L ^-1^)	1.40 ± 0.04	1.03 ± 0.04	1.24 ± 0.02	1.73 ± 0.02	0.33 ± 0.01	0.27 ± 0.01
Nitrate (μM L ^-1^)	10.51 ± 0.24	9.42 ± 0.20	15.22 ± 0.68	13.48 ± 0.32	4.99 ± 0.28	8.28 ± 1.44
SRP (μM L^-1^)	1.38 ± 0.33	1.77 ± 0.15	2.15 ± 0.07	2.88 ± 0.50	0.62 ± 0.26	1.81 ± 0.33

Values for salinity, temperature, pH, dissolved oxygen, nitrate, nitrite and SRP provided here represent average ± SD.

### Microscopic images

**Fig 1:**
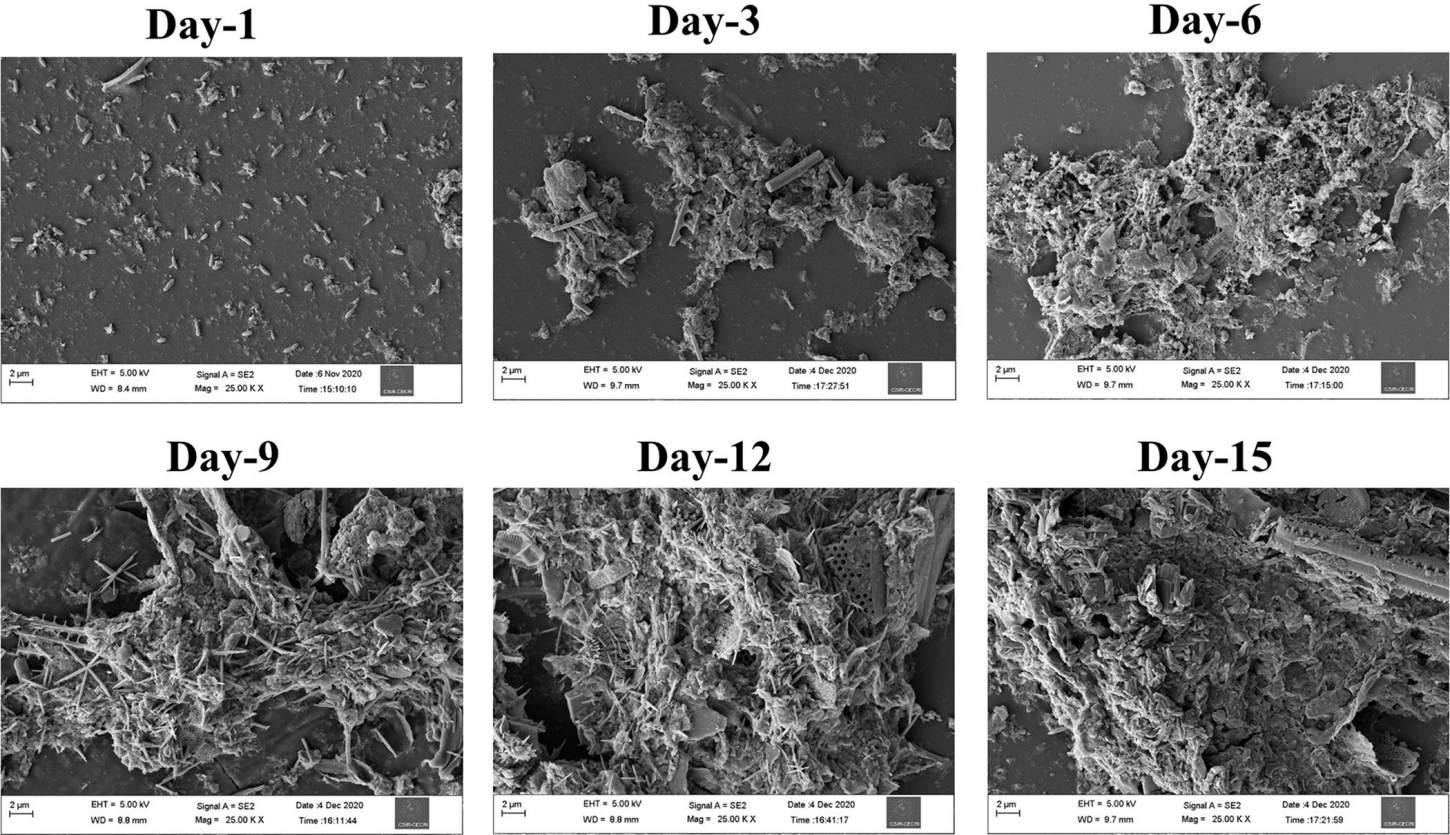
Representative field-emission scanning electron microscope (FESEM) images of acrylic coupons deployed in the intake area of a power plant located in the Laccadive Sea for different durations (1, 3, 6, 9, 12 and 15 days). Images display an increase in biofilm biomass and exopolymeric substance (EPS) with increasing time.

FESEM images depicting biofilms formation on acrylic coupons are shown (**Fig 1**). These micrographs revealed that the immersed substrata were colonized rapidly in the studied environment and therefore microbial colonization can be visualized within 1-day of immersion. In addition, a steady increase in biofilm biomass and content of extra polymeric substances (EPS) along the successional days 3, 6, 9, 12, and 15 were observed. After day-9, we observed a minor growth of macrofoulers such as tiny barnacles, mussels, and algae. The growth of lower eukaryotes viz. diatoms can also be seen in the microscopic images of 9, 12 and 15-day biofilms.

### Sequencing outputs

A total of 24 samples from both marine biofilms and seawater were selected to generate bacterial community data on microfouling succession using high-throughput tag sequencing. Of the selected samples, 18 samples (3 substrata × 6 sampling occasions) and 6 samples (1 sample × 6 sampling occasions) were from marine biofilms and seawater, respectively. Here, the characters used to define samples name denotes sampling days (e.g. day-1: 1d, day-3: 3d) and their sources (biofilm assembled on stainless steel: SS, biofilm assembled on high-density polyethylene: HP, biofilm assembled on titanium: Ti, and seawater: SW).

Sequencing of the V3-V4 region using Illumina MiSeq platform generated a total of 4,608,494 paired-end raw reads. As summarized (**S1 Table in S1 File)**, the read values were least for day-9 (536,986) and highest for day-12 (611,800). With regard to substrata, the number of reads was higher in samples of HP (1,168,714) and least in Ti (1,090,110). Assembly and applying of quality filtering criteria resulted in 1,496,022 reads, of which, 1,191,649 reads hit the reference. To equalize the uneven sequencing depth, all samples were rarefied to the lowest number of reads (35,998 of sample 1dSW). Finally, high-quality reads were clustered into 3,655 features with a range of 56–1167. The sparse curves with a cluster distance of 0.03 were saturated reaching asymptote indicating adequate sequencing depth per sample to cover the whole diversity of both sessile and planktonic samples (**S2 Fig in S1 File**).

### Bacterial diversity and community composition of biofilm-forming microbiota is determined by temporal succession than substrata materials

**Fig 2:**
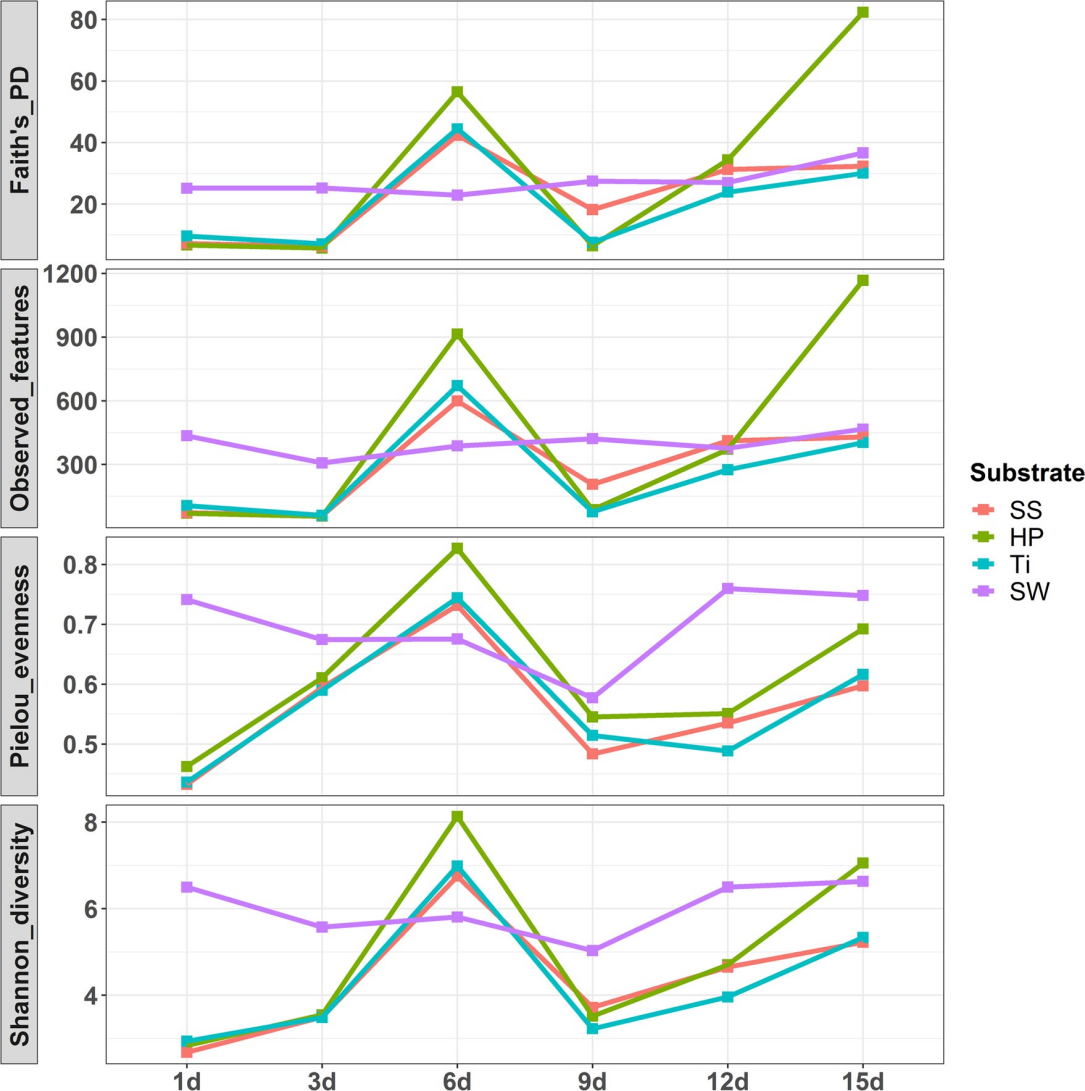
Alpha diversity indices of biofilm-forming and seawater-associated bacterial communities. Images displaying diversity, richness, and evenness of marine biofilms assembled on three artificial substrata viz., stainless steel (SS), titanium (Ti) and high-density polyethylene (HP) and peripheral seawater (SW) collected during day-1 to day-15 from the southern coastal region of India.

The Shannon diversity index–a measure that represents taxa and their distribution (i.e. evenly distributed or skewed), trended toward significance when comparing succession days (Kruskal–Wallis, H = 20.89, p = 0.001) but not substrate material (Kruskal–Wallis, H = 4.03, p = 0.25). Similarly, the results of Faith’s_PD revealed that the differences in bacterial richness among substrata types were significantly less (Kruskal–Wallis, H = 0.406, p = 0.938) as compared to the temporal succession pattern (Kruskal–Wallis, H = 19.7, p = 0.003) (**Fig 2 and S1 Table in S1 File**). The development of early-stage biofilm on substrate resulted in a continuous elevation in bacterial richness and diversity from the succession day-1 onwards and reached a maximum at day-6. Later, the biofilm development demonstrated a reduction in both richness and diversity from day-9, and thereafter it remained constant during day-12 and again rose at day-15. Highest number of features and greater evenness were observed during day-6 and day-15 (**Fig 2 and S1 Table in S1 File**). Regarding the effect of substrate type, the Shannon diversity was least in SS and higher in HP, while the bacterial richness was higher in SS and less in Ti. Nevertheless, there were no significant differences in diversity indices observed among the substrata types.

**Fig 3:**
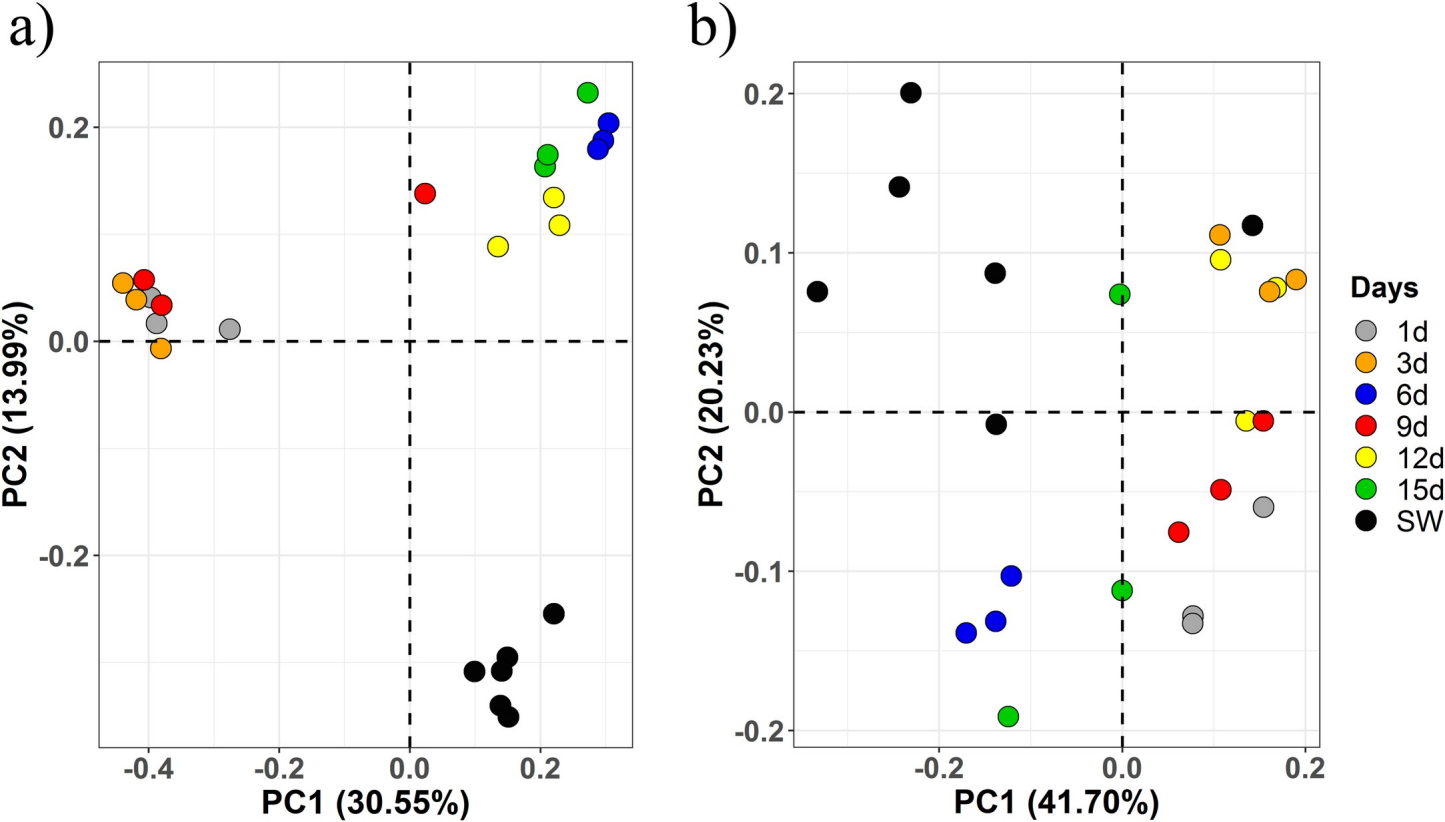
Principal coordinate analysis (PCoA) plots showing variation in biofilm-forming and seawater-associated bacterial communities collected for consecutively 15 days from the southern coastal region of India. a) PCoA plot displaying clustering pattern based on unweighted UniFrac (take presence/absence matrix in the account). b) PCoA plot displaying clustering pattern based on weighted UniFrac (take abundance in the account). Coloured circles in the PCoA plots represent the days of marine biofilms and seawater sampling.

Principal coordinate analysis (PCoA) based on both unweighted (presence-absence-based) and weighted (abundance-based) UniFrac distance was constructed to compare the whole microbiota structure of the early-stage biofilms (**Fig 3**). Obtained results of both PCoA displayed no clustering of samples based on the substrate type whereas, the clustering occurred on the basis of succession days (**Fig 3a and 3b**). Interestingly, we observed two major clusters: biofilm-forming communities of succession day-1, day-3 and day-9 were clustered distinctly into 1 group whereas, biofilm-forming communities of succession day-6, day-12, and day-15 as another on the unweighted UniFrac-based PCoA plot with a variance of 30.55% at axis1 (PC1) (**Fig 3a**). The significance in clustering of samples by days in PCoA was supported by a non-parametric multivariate analysis of variance (PERMANOVA, F = 4.077, p < 0.001). In the case of weighted UniFrac-based PCoA, biofilms of day-1, day-3, day-9 and day-12 were grouped as a single cluster leaving behind biofilms of day-6 and day-15 (PERMANOVA, F = 4.873, p < 0.001) (**Fig 3b**). It should be emphasized here that the community variations observed during various stages of biofilm development is higher based on the presence/absence of bacterial taxa rather than their abundance. Overall, these results indicated the negligible influence of substrata types whereas, strong influence of temporal succession in determining the early-stage microbiota.

### Impact of temporal succession remained more prominent than substrate types on taxon abundance of biofilm-forming microbiota

The Illumina-generated reads were next allocated at various taxonomic ranks to evaluate the temporal succession pattern of biofilm-forming microbiota. The relative proportions of dominant bacterial taxa are represented at phylum (**S3 Fig in S1 File**) and class (**S4 Fig in S1 File**) levels. At phylum and class levels, no visible difference of bacterial community composition between substrata types was observed throughout the succession stages. The bacterial community composition of the biofilms was largely dominated by the phylum *Proteobacteria* (92.78% ± 6.31%), followed by *Bacteroidetes* (5.61% ± 4.73). Other phyla such as *Cyanobacteria*, *Planctomycetes*, and *Firmicutes* constituted for < 2% of the overall biofilm-forming bacterial community. At the class level, the community composition was dominated by three major classes viz., *Gammaproteobacteria* (83.13 ± 13.77%), *Alphaproteobacteria* (6.66 ± 5.33%), and *Flavobacteriia* (5.25 ± 4.57%) (**S4 Fig in S1 File**).

**Fig 4:**
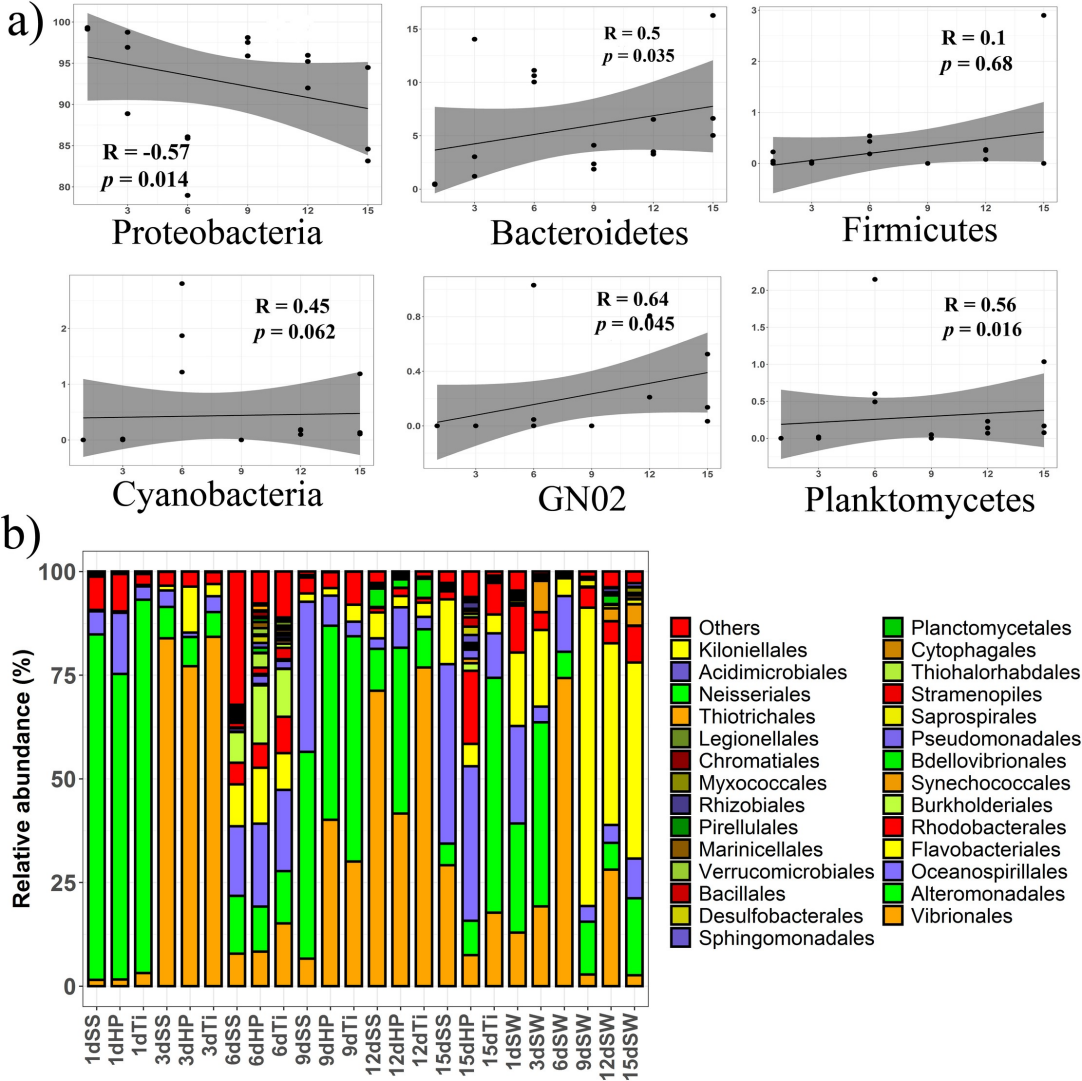
Temporal dynamics of the relative abundance of biofilm-forming bacterial communities. a) Spearman correlation plots of six dominant bacterial phyla over days. “R” represents the Spearman correlation coefficient. b) Stacked bar plots showing the relative abundance of dominant taxonomic groups at order level. Only those taxonomic groups that constitute for more than 0.5% of the total community are shown.

When we performed regression analysis with non-parametric Spearman correlation to understand the temporal pattern of dominant marine phyla, a significant positive linear association with succession days was observed for most of the phyla including *Bacteroidetes* (p < 0.05), *Cyanobacteria* (p < 0.05), *Planctomycetes* (p < 0.05), and *GN02* (p < 0.005) (**Fig 4a**). Contrastingly, phylum *Proteobacteria* showed a strong and significant (p < 0.05) negative linear correlation along the succession days.

**Fig 5:**
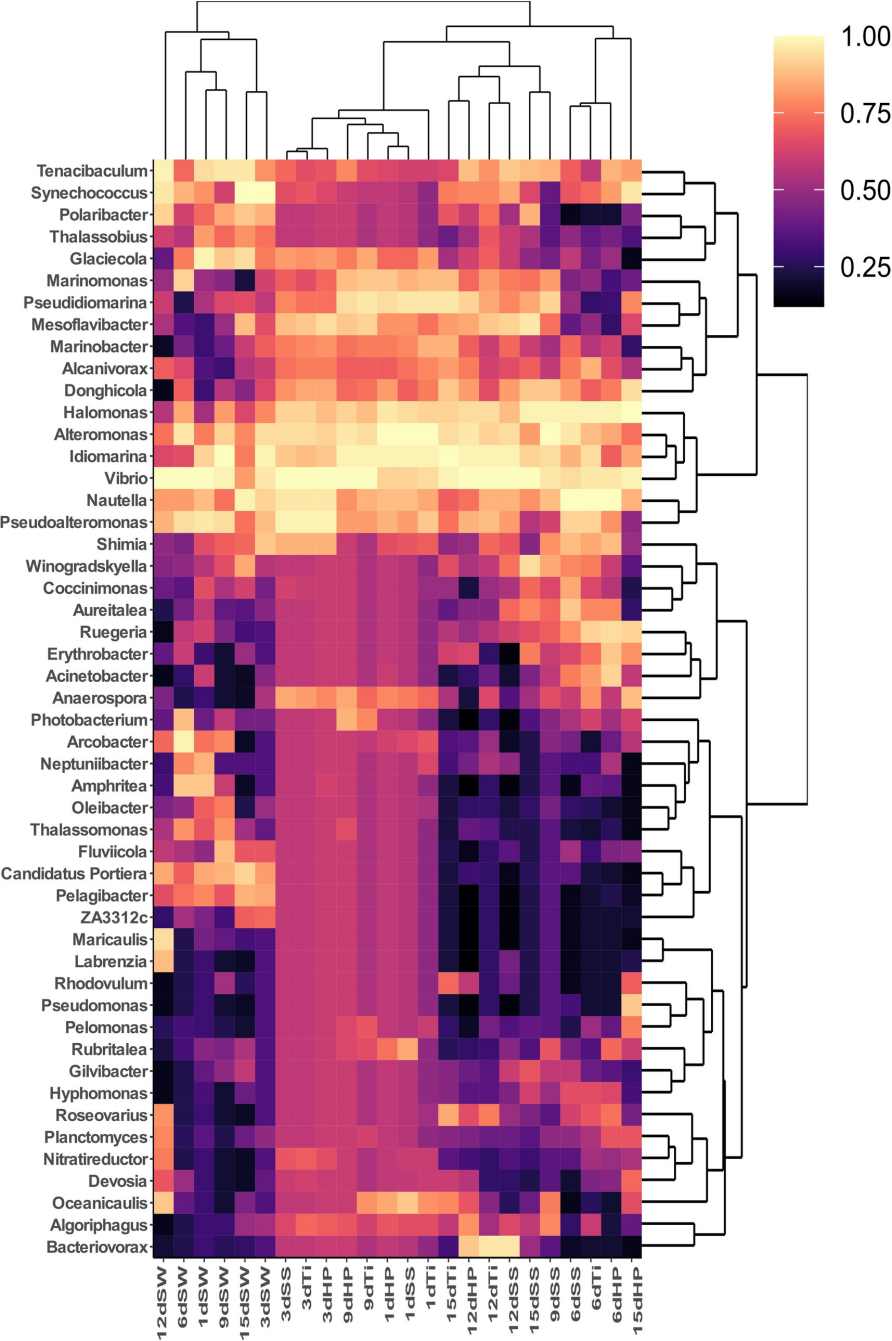
Hierarchically clustered heatmap analysis of the bacterial community composition in the biofilm and seawater samples collected on different days. Figure shows the clustering of seawater samples separately from the biofilm samples and the colours represent the relative abundance of the top abundant 50 genera.

The study on the pattern of temporal succession became more interesting and visible when the relative abundance was analyzed at the order level (**Fig 4b**), where every individual succession day was characterized by unique taxonomic profile. For example, the relative abundance of *Alteromonadales* tended to be significantly (p < 0.005) higher during succession day-1 and day-9, while *Vibrionales* substantially (p < 0.005) dominated the biofilm-forming microbiota of succession day-3 and day-12. On the succession day-6 and day-15, a diverse numbers of bacterial orders were observed as compared to other succession days. *Oceanospirillales* was the third abundant bacterial order that was found proportionally high during succession day-6 and day-15. Interestingly, when significantly differed families among succession days were analyzed using STAMP software, we observed that there are nine families that can be categorized into early and late biofilm formers (**S5 Fig in S1 File**). For example, families *Rhodobacteraceae* (day-3 and day-6) and *Pseudoalteromonadaceae* (day-1 and day-9) were found to be significantly abundant during the initial days of biofilm while, families such as *Verrucomicrobiaceae*, *Sinabactereraceae*, *Cryomorphaceae* were abundant during later six days (day-12 and-15) of biofilm development. Similar to higher taxonomic ranks, the community composition difference was clearly visible when we looked at the genus level (**Fig 5**). We observed that genera *Vibrio*, *Alteromonas*, *Idiomarina*, *Halomonas* and *Pseudoalteromonas* were dominant groups among the biofilm samples. On the other hand, biofilms developed on the three artificial substrata displayed no significant variations in taxon abundance, further highlighting the unexpected result of the negligible influence of substrate type on biofilm-forming microbiota.

LEfSe analysis was performed for identifying biomarker taxa associated with succession days and substrate types. This analysis revealed that except for the day-9 and day-12, biofilm-forming microbiota of all other succession days was dominated by specific bacterial taxa with day-6 being the most abundant, followed by day-15 (**S6 Fig in S1 File**). Most prominently, abundant orders such as *Alteromonadales* and *Vibrionales* were identified as biomarkers for day-1 and day-3, respectively. Similarly, other bacterial orders viz., *Rhodobacterales*, *Stramenophiles*, *Sphingomonadales* and *Campylobacterales* were exclusive for day-6 and *Rhizhobiales* and *Synechococcales* were exclusive for day-15. Contrary to the succession days, no substrate type-specific bacterial taxa were identified.

### Variations in biofilm-forming microbiota is independent of the community composition of seawater

**Fig 6:**
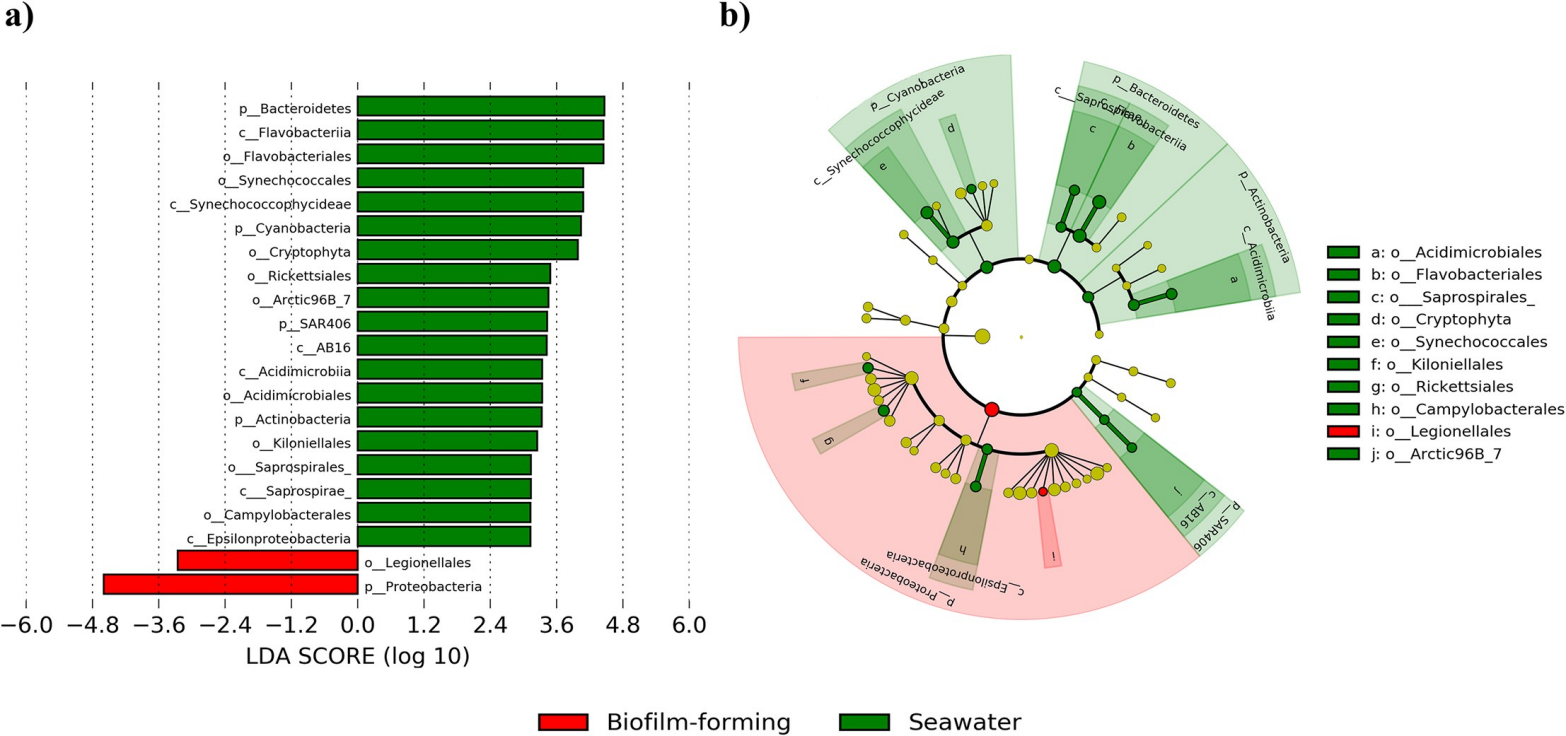
Bar plot and cladogram depicting the results of LEfSe analysis that identified the significantly differed taxa between biofilm and seawater bacterial community composition. Bacterial taxa with linear discriminant analysis (LDA) score greater than 3 are displayed in the figures.

To further study the personalized community composition of biofilms, the similarity between seawater (collected during all sampling occasions) and biofilm-forming microbiota was investigated. As can be seen in PCoA plots, the dissimilarity between the microbiota of seawater and biofilms was the most pronounced than the differences between temporal succession and substrata types. In the unweighted UniFrac-based PCoA plot, the microbiota composition of all planktonic community (SW samples) were evidently separated from the biofilm microbiota with 30.55% variation in PC1 and 13.99% variation in PC2 (**Fig 3a**). However, the weighted UniFrac ordination analysis showed some overlapping of SW samples with biofilm-forming bacterial communities but the variation observed was comparatively high (41.70% in and 20. 23% in PC2) (**Fig 3b**). These results were supported by highest significance (p < 0.002) in PERMANOVA analysis. Similarly, when the distance of dissimilarity is considered, succession day-1 and day-3 were more apart from the seawater samples. The observations made in the PCoA ordination plots was further strengthened by UPGMA analysis (**S7 Fig in S1 File**) and heatmap clustering at the genus level (**Fig 5**). These results further confirmed that biofilm-forming bacterial community composition is characterized by distinct groups of bacterial genera as compared to surrounding free-living bacterial communities. The differences in the relative proportions of dominant bacterial genera such as *Alteromonas*, *Idiomarina*, *Halomonas*, *Pseudidiomarina*, *Vibrio*, *Pseudoalteromonas*, *Polaribacter*, and *Glaciecola* contributed profoundly to the observed differences. In support of these outcomes, LEfSe analysis revealed that both high- and low-abundance taxa differed significantly between biofilm-forming and seawater bacterial communities. Phylum *Proteobacteria* was enriched in biofilm-forming microbiota, whereas *Bacteroidetes*, *Cyanobacteria*, *SAR406*, and *Actinobacteria* were abundant in seawater (**Fig 6**). Collectively, these results reinforce a general notion that there exist particular bacterial communities that can only adhere and proliferate successfully to form biofilms in marine environment. Not all the taxa present in seawater are able to adhere and form biofilms.

## Discussion

Microbial colonization on submerged surfaces is a prime phenomenon in aquatic ecosystems. The structure and community composition of biofouling development are largely determined by microbial recruitment from the planktonic (free-floating) community, and the mechanism of pioneering succession determines the stability of the long-term biofouling assemblages [51–54]. Several factors including the environmental heterogeneity, substrate material, spatio-temporal variations can influence the recruitment of species [52, 55, 56], and understanding these key associations provide useful insights into their control strategies. Extending on our previous research work [57], the present study describes the bacterial community succession that occurs during the early stages of biofilm development on various artificial substrata.

Succession of biofilm-forming bacterial community formed on various artificial surfaces has been studied extensively [8, 19, 26, 30–32, 51], with higher diversity observed during the first 24 h of immersion. Our findings are relatively different from this description as we observed greater diversity on succession day-6 and day-15. A significant increase in Shannon diversity index during succession day-3 and day-6 and between succession day-12 and day-15 might be driven by an increase in evenness. However, the changes were insignificant at the substrate level. Similarly, Faith’s PD also showed a significant increase between succession day-3 and day-6 and day-12 and day-15 as an effect of recruitment of new and phylogenetically distinct bacterial taxa during the development of biofilm. The high richness and low evenness on succession day-1, 9, and day-3, 12 can be explained by the presence of dominant taxa such as *Alteromonadales* and *Vibrionales*, respectively. Moreover, the dominance of competitive species may also be the reason behind lower diversity during the initial days of biofilm development [23, 58, 59].

In parallel with several earlier studies, the pioneering biofilm-forming community was largely dominated by *Gammaproteobacteria* particularly the order *Alteromonadales*. The members of *Alteromonadales* are frequently reported as an important primary colonizers of submerged substrata and are found to be dominant in the young biofilm [4, 17, 21] as they are widely involved in recruitment of subsequent micro- and macro-foulers [21, 60]. Members of *Alteromonadales* are also known to protect the biofilms from land-based contaminants [7]. Similarly, few other orders such as *Vibrionales*, *Oceanospirillales*, and *Pseudomonadales* also accompanied *Alteromonadales* in establishing early-stage biofilm. These bacterial orders are identified as prominent members to cause biocorrosion on steel surfaces [61]. Few assumptions have been proposed to account for the high prevalence of *Gammaproteobacteria* at the early-stage biofilm development, which includes the presence of diversified members in this class with their ability to thrive in various environmental conditions [21, 23].

Contrary to this, few studies [17, 23, 30, 31] have reported *Alphaproteobacteria* (members of *Rhodobacteraceae*) as a prominent member of the early-stage (developed within 24–48 h) biofilm community, which could be basically due to differences in environmental conditions. However, we also observed a significantly higher abundance of family *Rhodobacteraceae* during day-3 and day-6, which is in accordance with Dang and Lovell, (2002) [29].

Moving onto the taxon abundance, we witnessed that the relative abundances of dominant bacterial taxa change in a continuum from succession day-1 over the entire succession period. We also observed a recurrence of the same pattern change with two major shifts in the bacterial community composition. First shift was observed beyond 24 h of succession, where the community dominance shifted from *Alteromonadales* (members of *Alteromonadaceae* and *Idiomarinaceae*) to *Vibrionales* (members of *Vibrionaceae*). The second shift occurred between succession day-3 and day-6, wherein the community composition of the members of *Oceanospirallales*, *Flavobacteriales*, *Rhodobacterales*, *Bhurkholderiales*, *Pseudomonadales* became equally proportional to that of *Alteromonadales* and *Vibrionales*. These dominant members are also witnessed as biomarkers of the respective days in LEfSe analysis. The same pattern of compositional shift observed during succession day-1 to day-6 was repeated for the rest of the sampling days, wherein, the diverse biofilm-forming microbiota of succession day-6 reduced again at day-9 during which the dominance of *Alteromonadales* prevailed. Following this, *Vibrionales* dominated the biofilm microbiota of succession day-12 as similar to succession day-3. At succession day-15, the community composition was once again diversified.

Generally, all the successional communities display an increase in diversity and richness in their early-stage development, which makes it difficult to explain the results of our observation on pattern of reduction and increase in diversity. A lake-based wetland mesocosm research by Jackson et al. also noted a similar nature of variations, explaining that it would have been possibly due to the competitive dominance of some bacterial populations [59]. This might also be a result of the transitory phase of maximum richness in the middle stages of community growth, similar to what the intermediate disruption hypothesis suggests [62]. However, this pattern of entire community shift has been rarely documented by any of the previous studies, which may be due to the fact that most of these studies have discussed the change in succession at higher taxonomic “phylum and/or class” [7, 8, 27, 63] or lower taxonomic “genus” [26, 28] levels, where either dominance or diversity prevails, respectively. This shift was keenly observed at the order level.

At the level of substrata, our experimental setup on the succession pattern revealed that the substrate type was not as influential as the succession days. Generally, it has been shown that the bacterial attachment in marine environment is highly influenced by the physico-chemical properties of the surfaces [27, 64, 65] and hence the substrata types have been frequently reported to determine the bacterial community composition of marine biofilms [17, 26]. Our findings refuted the presumption that substrate material has a major impact on early-stage of marine biofilm formation; rather, the effect of substrate material was overwhelmed by temporal succession and *in situ* environmental conditions. However, HDPE demonstrated greater diversity than Ti and SS, owing to the surface roughness and hydrophobicity of SS and the toxicity of Ti [26]. Nevertheless, these differences were not statistically significant in comparison with the observed differences in temporal succession. In line with this, Bellou et al. and Lee et al. also noticed the importance of environment, rather than substrata types, in defining the biofilm community structure in the deep sea [27, 28].

We also observed a clear difference between biofilm-forming and planktonic bacterial community composition. Planktonic communities characterized consistently by heterogeneous taxonomic groups and higher diversity than biofilm-forming communities [23]. Ecologically, the principle that “everything is everywhere”, and the environment selects do apply in the case of species sorting and biofilm development [10, 66]. Higher diversity and community composition in seawater may be surmised by the fact that all microorganisms in the ocean are widely distributed, but majority of the species are only latent in a given environment. We identified several bacterial groups, whose abundance was observed solely either to the planktonic (e.g. *Bacteroidetes*, *Cyanobacteria*) or attached state (e.g. *Proteobacteria*, *Legionellales*) causing overall community differences. These results suggest that there exist a stronger influence of species sorting in structuring biofilm-forming microbiota in the studied marine environment, which is in agreement with the previous studies focusing on marine biofilms assembled on different artificial substrates in cold seep systems [26, 28]. Moreover, we also identified an order *Legionellales* that was overrepresented in biofilm-forming microbiota. Members of *Legionellales* are considered as predominant members associated with water systems of built environment and are potent parasites [67]. Their existence in the biofilm-forming microbiota may have been enhanced by the presence of other organisms besides bacteria such as amoeba [68]. Apart from these, there were more biomarkers associated with planktonic part than biofilms suggesting that several bacterial groups in marine environment are adapted solely either to the planktonic or attached state causing overall community differences. Perhaps, changing the environmental conditions and parameters might contribute higher diversity to biofilm microbiota.

In conclusion, our findings revealed a distinct ecological succession in the early stages of marine biofilm. Although several studies compared the bacterial assembly on artificial substrata, this is the first pilot study showing the early-stage bacterial biofilm succession in the coastal region of India. Moreover, since our prime target was on the substrata that are widely used in coastal located industries, specifically power plants, we limited the substrata types to three (SS, HP, and Ti). Higher variations in substrate material and their replicate diversity analysis may result in some significant changes to support the theory of species sorting with respect to substrata types. Nevertheless, based on the obtained data in this study, we conclude that the *in situ* environment and temporal succession play a major role in structuring early-stage biofilm microbiota in the southern coastal seawater of India. Analyzing the succession pattern of macrofouling communities in future studies may shed more light on the role of a particular bacterial community on a single substratum.

## Supporting information

S1 File Supporting figures and tables. This PDF file contains (1) S1 Fig. Setup employed for the development of biofilm in the intake area of a coastal power plant located in the southern coastal region of India. (2) S2 Fig. Rarefaction curves displaying the observed features with an increasing number of reads. (3) S3 Fig. Taxonomic composition and abundance distribution of biofilm-forming and seawater bacterial communities at the phylum level. (4) S4 Fig. Taxonomic classification of biofilm-forming and seawater bacterial communities at the class level over different days. (5) S5 Fig. Bar plots with extended errors displaying the proportional differences of the significantly differed bacterial taxa among the succession days at family level. (6) S6 Fig. LEfSe results displaying the significantly differed bacterial order among the succession days. (7) S7 Fig. Similarity of microbial communities from the biofilm and seawater samples, as illustrated by UPGMA hierarchical clustering and (8) S1 Table. Glimpse of Illumina-generated reads before and after applying quality control criteria and values of alpha diversity indices.
(PDF)

## References

[1] Flemming HC, Wingender J. The biofilm matrix. Nat Rev Microbiol. 2010;8(9):623–33. doi: 10.1038/nrmicro2415 20676145

[2] Doghri I, Rodrigues S, Bazire A, Dufour A, Akbar D, Sopena V, et al. Marine bacteria from the French Atlantic coast displaying high forming-biofilm abilities and different biofilm 3D architectures. BMC Microbiol. 2015;15(1):1–10. doi: 10.1186/s12866-015-0568-4 26498445 PMC4619314

[3] Costerton JW, Stewart PS, Greenberg EP. Bacterial biofilms: a common cause of persistent infections. Science. 1999;284(5418):1318–22. doi: 10.1126/science.284.5418.1318 10334980

[4] Lee JW, Nam JH, Kim YH, Lee KH, Lee DH. Bacterial communities in the initial stage of marine biofilm formation on artificial surfaces. J Microbiol. 2008;46(2):174–82. doi: 10.1007/s12275-008-0032-3 18545967

[5] Wahl M, Goecke F, Labes A, Dobretsov S, Weinberger F. The second skin: ecological role of epibiotic biofilms on marine organisms. Front Microbiol. 2012;3:292. doi: 10.3389/fmicb.2012.00292 22936927 PMC3425911

[6] Karatan E, Watnick P. Signals, regulatory networks, and materials that build and break bacterial biofilms. Microbiol Mol Biol Rev. 2009;73(2):310–47. doi: 10.1128/MMBR.00041-08 19487730 PMC2698413

[7] Rampadarath S, Bandhoa K, Puchooa D, Jeewon R, Bal S. Early bacterial biofilm colonizers in the coastal waters of Mauritius. Electron J Biotechnol. 2017;29:13–21. 10.1016/j.ejbt.2017.06.006

[8] Pollet T, Berdjeb L, Garnier C, Durrieu G, Le Poupon C, Misson B, et al. Prokaryotic community successions and interactions in marine biofilms: the key role of *Flavobacteriia*. FEMS Microbiol Ecol. 2018;94(6):fiy083. doi: 10.1093/femsec/fiy083 29733333

[9] Salta M, Wharton JA, Blache Y, Stokes KR, Briand JF. Marine biofilms on artificial surfaces: structure and dynamics. Environ Microbiol. 2013;15(11):2879–93. doi: 10.1111/1462-2920.12186 23869714

[10] Zhang W, Ding W, Li YX, Tam C, Bougouffa S, Wang R, et al. Marine biofilms constitute a bank of hidden microbial diversity and functional potential. Nat Commun. 2019;10(1):1–10. doi: 10.1038/s41467-018-07882-8 30705275 PMC6355793

[11] Saravanan P, Priya AM, Sundarakrishnan B, Venugopalan VP, Rao TS, Jayachandran S. Effects of thermal discharge from a nuclear power plant on culturable bacteria at a tropical coastal location in India. J Therm Biol. 2008;33(7):385–94. 10.1016/j.jtherbio.2008.06.006

[12] Rajeev M, Sushmitha TJ, Prasath KG, Toleti SR, Pandian SK. Systematic assessment of chlorine tolerance mechanism in a potent biofilm-forming marine bacterium *Halomonas boliviensis*. Int Biodeterior Biodegradation. 2020;151:104967. 10.1016/j.ibiod.2020.104967

[13] Choi DH, Noh JH, Yu OH, Kang YS. Bacterial diversity in biofilms formed on condenser tube surfaces in a nuclear power plant. Biofouling. 2010;26(8):953–9. doi: 10.1080/08927014.2010.533267 21058056

[14] Ludensky M. Control and monitoring of biofilms in industrial applications. Int Biodeterior Biodegradation. 2003;51(4):255–63. 10.1016/S0964-8305(03)00038-6

[15] Koch GH, Brongers MP, Thompson NG, Virmani YP, Payer JH. Cost of corrosion in the United States. In Handbook of environmental degradation of materials. William Andrew Publishing; 2005. pp. 3–24. 10.1016/B978-081551500-5.50003-3

[16] Walker ME, Safari I, Theregowda RB, Hsieh MK, Abbasian J, Arastoopour H, et al. Economic impact of condenser fouling in existing thermoelectric power plants. Energy. 2012;44(1):429–37. 10.1016/j.energy.2012.06.010

[17] Dang H, Lovell CR. Bacterial primary colonization and early succession on surfaces in marine waters as determined by amplified rRNA gene restriction analysis and sequence analysis of 16S rRNA genes. Appl Environ Microbiol. 2000;66(2):467–75. doi: 10.1128/AEM.66.2.467-475.2000 10653705 PMC91850

[18] Briand JF, Djeridi I, Jamet D, Coupé S, Bressy C, Molmeret M, et al. Pioneer marine biofilms on artificial surfaces including antifouling coatings immersed in two contrasting French Mediterranean coast sites. Biofouling. 2012;28(5):453–63. doi: 10.1080/08927014.2012.688957 22582937

[19] Briand JF, Barani A, Garnier C, Réhel K, Urvois F, LePoupon C, et al. Spatio-temporal variations of marine biofilm communities colonizing artificial substrata including antifouling coatings in contrasted French coastal environments. Microb Ecol. 2017;74(3):585–98. doi: 10.1007/s00248-017-0966-2 28374061

[20] Elifantz H, Horn G, Ayon M, Cohen Y, Minz D. *Rhodobacteraceae* are the key members of the microbial community of the initial biofilm formed in Eastern Mediterranean coastal seawater. FEMS Microbiol Ecol. 2013;85(2):348–57. doi: 10.1111/1574-6941.12122 23551015

[21] Lawes JC, Neilan BA, Brown MV, Clark GF, Johnston EL. Elevated nutrients change bacterial community composition and connectivity: high throughput sequencing of young marine biofilms. Biofouling. 2016;32(1):57–69. doi: 10.1080/08927014.2015.1126581 26751559

[22] Muthukrishnan T, Al Khaburi M, Abed RM. Fouling microbial communities on plastics compared with wood and steel: are they substrate-or location-specific?. Microb Ecol. 2019;78(2):361–74. doi: 10.1007/s00248-018-1303-0 30535914

[23] Antunes JT, Sousa AG, Azevedo J, Rego A, Leão PN, Vasconcelos V. Distinct temporal succession of bacterial communities in early marine biofilms in a Portuguese Atlantic Port. Front Microbiol. 2020;11:1938. doi: 10.3389/fmicb.2020.01938 32849482 PMC7432428

[24] Langenheder S, Székely AJ. Species sorting and neutral processes are both important during the initial assembly of bacterial communities. ISME J. 2011;5(7):1086–94. doi: 10.1038/ismej.2010.207 21270841 PMC3146284

[25] Meier A, Tsaloglou NM, Mowlem MC, Keevil CW, Connelly DP. Hyperbaric biofilms on engineering surfaces formed in the deep sea. Biofouling. 2013;29(9):1029–42. doi: 10.1080/08927014.2013.824967 23964799

[26] Zhang WP, Wang Y, Tian RM, Bougouffa S, Yang B, Cao HL, et al. Species sorting during biofilm assembly by artificial substrates deployed in a cold seep system. Sci Rep. 2014;4(1):1–7. 10.1038/srep06647 PMC4200420 25323200

[27] Bellou N, Papathanassiou E, Dobretsov S, Lykousis V, Colijn F. The effect of substratum type, orientation and depth on the development of bacterial deep-sea biofilm communities grown on artificial substrata deployed in the Eastern Mediterranean. Biofouling. 2012;28(2):199–213. doi: 10.1080/08927014.2012.662675 22352335

[28] Lee OO, Wang Y, Tian R, Zhang W, Shek CS, Bougouffa S, et al. *In situ* environment rather than substrate type dictates microbial community structure of biofilms in a cold seep system. Sci Rep. 2014;4(1):1–10. 10.1038/srep03587 PMC5378041 24399144

[29] Dang H, Lovell CR. Numerical dominance and phylotype diversity of marine *Rhodobacter* species during early colonization of submerged surfaces in coastal marine waters as determined by 16S ribosomal DNA sequence analysis and fluorescence *in situ* hybridization. Appl Environ Microbiol. 2002;68(2):496–504. doi: 10.1128/AEM.68.2.496-504.2002 11823183 PMC126732

[30] Jones PR, Cottrell MT, Kirchman DL, Dexter SC. Bacterial community structure of biofilms on artificial surfaces in an estuary. Microb Ecol. 2007;53(1):153–62. doi: 10.1007/s00248-006-9154-5 17186146

[31] Dang H, Li T, Chen M, Huang G. Cross-ocean distribution of *Rhodobacterales* bacteria as primary surface colonizers in temperate coastal marine waters. Appl. Environ. Microbiol. 2008;74(1):52–60. doi: 10.1128/AEM.01400-07 17965206 PMC2223210

[32] D’ambrosio L, Ziervogel K, MacGregor B, Teske A, Arnosti C. Composition and enzymatic function of particle-associated and free-living bacteria: a coastal/offshore comparison. ISME J. 2014;8(11):2167–79. doi: 10.1038/ismej.2014.67 24763371 PMC4992077

[33] Abed RM, Al Fahdi D, Muthukrishnan T. Short-term succession of marine microbial fouling communities and the identification of primary and secondary colonizers. Biofouling. 2019;35(5):526–40. doi: 10.1080/08927014.2019.1622004 31216872

[34] Lakshmi K, Muthukumar T, Doble M, Vedaprakash L, Dineshram R, Jayaraj K, et al. Influence of surface characteristics on biofouling formed on polymers exposed to coastal sea waters of India. Colloids Surf B Biointerfaces. 2012;91:205–11. doi: 10.1016/j.colsurfb.2011.11.003 22143025

[35] Palanichamy S, Subramanian G, Eashwar M. Corrosion behaviour and biofouling characteristics of structural steel in the coastal waters of the Gulf of Mannar (Bay of Bengal), India. Biofouling. 2012;28(5):441–51. doi: 10.1080/08927014.2012.684947 22554304

[36] Jayachandran PR, Jima M, Philomina J, Sanu VF, Nandan SB. Invasion of biofouling mussel *Mytilopsis Conrad*, 1857 (*Bivalvia: Dreissenacea*) in the Cochin backwaters, southwest coast of India. Curr Sci. 2018;115(12):2198–200.

[37] Khan MF, Wesley SG. Assessment of health safety from ingestion of natural radionuclides in seafoods from a tropical coast, India. Mar Pollut Bull. 2011;62(2):399–404. doi: 10.1016/j.marpolbul.2010.12.016 21251682

[38] Satheesh S, Wesley SG. Seasonal changes of motile polychaetes in the fouling assemblage developed on test panels submerged on a tropical coast. Marine Biological Association of the United Kingdom. J Mar Biolog Assoc U.K. 2013;93(6):1525. 10.1017/S0025315413000076

[39] Carpenter JH. The Chesapeake Bay Institute technique for the Winkler dissolved oxygen method. Limnol Oceanogr. 1965;10(1):141–3. 10.4319/lo.1965.10.1.0141

[40] Parsons TR. A manual of chemical & biological methods for seawater analysis. Elsevier; 2013.

[41] García-Robledo E, Corzo A, Papaspyrou S. A fast and direct spectrophotometric method for the sequential determination of nitrate and nitrite at low concentrations in small volumes. Mar Chem. 2014;162:30–6. 10.1016/j.marchem.2014.03.002

[42] Rajeev M, Sushmitha TJ, Aravindraja C, Toleti SR, Pandian SK. Exploring the impacts of heavy metals on spatial variations of sediment-associated bacterial communities. Ecotoxicol Environ Saf. 2021;209:111808. doi: 10.1016/j.ecoenv.2020.111808 33360289

[43] Rajeev M, Sushmitha TJ, Aravindraja C, Toleti SR, Pandian SK. Thermal discharge‑induced seawater warming alters richness, community composition and interactions of bacterioplankton assemblages in a coastal ecosystem. Sci Rep. 2021; 11:17341. doi: 10.1038/s41598-021-96969-2 34462511 PMC8405676

[44] Takahashi S, Tomita J, Nishioka K, Hisada T, Nishijima M. Development of a prokaryotic universal primer for simultaneous analysis of Bacteria and Archaea using next-generation sequencing. PLoS ONE. 2014;9(8):e105592. doi: 10.1371/journal.pone.0105592 25144201 PMC4140814

[45] Bolyen E, Rideout JR, Dillon MR, Bokulich NA, Abnet CC, Al-Ghalith GA, et al. Reproducible, interactive, scalable and extensible microbiome data science using QIIME 2. Nat Biotechnol. 2019;37(8):852–7. doi: 10.1038/s41587-019-0209-9 31341288 PMC7015180

[46] Callahan BJ, McMurdie PJ, Rosen MJ, Han AW, Johnson AJ, Holmes SP. DADA2: high-resolution sample inference from Illumina amplicon data. Nat Methods. 2016;13(7):581–3. doi: 10.1038/nmeth.3869 27214047 PMC4927377

[47] Katoh K, Misawa K, Kuma KI, Miyata T. MAFFT: a novel method for rapid multiple sequence alignment based on fast Fourier transform. Nucleic Acids Res. 2002;30(14):3059–66. doi: 10.1093/nar/gkf436 12136088 PMC135756

[48] Price MN, Dehal PS, Arkin AP. FastTree 2–approximately maximum-likelihood trees for large alignments. PloS ONE. 2010;5(3):e9490. doi: 10.1371/journal.pone.0009490 20224823 PMC2835736

[49] DeSantis TZ, Hugenholtz P, Larsen N, Rojas M, Brodie EL, Keller K, et al. Greengenes, a chimera-checked 16S rRNA gene database and workbench compatible with ARB. Appl Environ Microbiol. 2006;72(7):5069–72. doi: 10.1128/AEM.03006-05 16820507 PMC1489311

[50] Segata N, Izard J, Waldron L, Gevers D, Miropolsky L, Garrett WS, et al. Metagenomic biomarker discovery and explanation. Genome Biol. 2011;12(6):1–8. doi: 10.1186/gb-2011-12-6-r60 21702898 PMC3218848

[51] Rao TS. Temporal variations in an estuarine biofilm: with emphasis on nitrate reduction. Estuar Coast Shelf Sci. 2003;58(1):67–75. 10.1016/S0272-7714(03)00060-X

[52] Rao TS. Comparative effect of temperature on biofilm formation in natural and modified marine environment. Aquat Ecol.2010;44(2):463–78. 10.1007/s10452-009-9304-1

[53] Sams MA, Keough MJ. Contrasting effects of variable species recruitment on marine sessile communities. Ecology. 2012;93(5):1153–63. doi: 10.1890/11-1390.1 22764501

[54] Brown NE, Milazzo M, Rastrick SP, Hall‐Spencer JM, Therriault TW, Harley CD. Natural acidification changes the timing and rate of succession, alters community structure, and increases homogeneity in marine biofouling communities. Glob Change Biol. 2018;24(1):e112–27. 10.1111/gcb.13856 28762601

[55] Cifuentes M, Krueger I, Dumont CP, Lenz M, Thiel M. Does primary colonization or community structure determine the succession of fouling communities?. J Exp Mar Biol Ecol. 2010;395(1–2):10–20. 10.1016/j.jembe.2010.08.019

[56] Logue JB, Lindström ES. Species sorting affects bacterioplankton community composition as determined by 16S rDNA and 16S rRNA fingerprints. ISME J. 2010;4(6):729–38. doi: 10.1038/ismej.2009.156 20130658

[57] Rajeev M, Sushmitha TJ, Toleti SR, Pandian SK. Culture dependent and independent analysis and appraisal of early stage biofilm-forming bacterial community composition in the Southern coastal seawater of India. Sci Total Environ. 2019;666:308–20. doi: 10.1016/j.scitotenv.2019.02.171 30798240

[58] Grime JP. Competitive exclusion in herbaceous vegetation. Nature. 1973;242(5396):344–7. 10.1038/242344a0

[59] Jackson CR, Churchill PF, Roden EE. Successional changes in bacterial assemblage structure during epilithic biofilm development. Ecology. 2001;82(2):555–66. 10.1890/0012-9658(2001)082[0555:SCIBAS]2.0.CO;2

[60] Lau SC, Mak KK, Chen F, Qian PY. Bioactivity of bacterial strains isolated from marine biofilms in Hong Kong waters for the induction of larval settlement in the marine polychaete *Hydroides elegans*. Mar Ecol Prog Ser. 2002;226:301–10. 10.3354/meps226301

[61] Xu J, Tang W, Ma J, Wang H. Comparison of microbial community shifts in two parallel multi-step drinking water treatment processes. Appl Microbiol Biotechnol. 2017;101(13):5531–41. doi: 10.1007/s00253-017-8258-9 28396926

[62] Connell JH. Diversity in tropical rain forests and coral reefs. Science. 1978;199(4335):1302–10. doi: 10.1126/science.199.4335.1302 17840770

[63] Guo Z, Wang L, Cong W, Jiang Z, Liang Z. Comparative Analysis of the Ecological Succession of Microbial Communities on Two Artificial Reef Materials. Microorganisms. 2021;9(1):120. doi: 10.3390/microorganisms9010120 33419197 PMC7825563

[64] Dexter SC, Sullivan JD, Williams JI, Watson SW. Influence of substrate wettability on the attachment of marine bacteria to various surfaces. Appl Microbiol. 1975;30(2):298–308. doi: 10.1128/am.30.2.298-308.1975 16350027 PMC187171

[65] Pinto M, Langer TM, Hüffer T, Hofmann T, Herndl GJ. The composition of bacterial communities associated with plastic biofilms differs between different polymers and stages of biofilm succession. PloS ONE. 2019;14(6):e0217165. doi: 10.1371/journal.pone.0217165 31166981 PMC6550384

[66] De Wit R, Bouvier T. ‘Everything is everywhere, but, the environment selects’; what did Baas Becking and Beijerinck really say?. Environ Microbiol. 2006;8(4):755–8. doi: 10.1111/j.1462-2920.2006.01017.x 16584487

[67] Bennett S, Bentham R. Effects of Seawater Concentration and Temperature on the Survival of *Legionella pneumophila* Serogroup 1. Legionella: State of the Art 30 Years after Its Recognition. 2006;420–2. 10.1128/9781555815660.ch99

[68] Gast RJ, Moran DM, Dennett MR, Wurtsbaugh WA, Amaral-Zettler LA. Amoebae and *Legionella pneumophila* in saline environments. J Water Health. 2011;9(1):37–52. doi: 10.2166/wh.2010.103 21301113 PMC3109871

