# Supplementary material for: Bacterial community structure of early-stage biofilms is dictated by temporal succession rather than substrate types in the southern coastal seawater of India
Source: PLoS One. 2021 Sep 27;16(9):e0257961. doi: 10.1371/journal.pone.0257961 (PMC8476003; doi:10.1371/journal.pone.0257961)
Supplement: S1 File — This PDF file contains (1) S1 Fig. Setup employed for the development of biofilm in the intake area of a coastal power plant located in the southern coastal region of India. (2) S2 Fig. Rarefaction curves displaying the observed features with an increasing number of reads. (3) S3 Fig. Taxonomic composition and abundance distribution of biofilm-forming and seawater bacterial communities at the phylum level. (4) S4 Fig. Taxonomic classification of biofilm-forming and seawater bacterial communities at the class level over different days. (5) S5 Fig. Bar plots with extended errors displaying the proportional differences of the significantly differed bacterial taxa among the succession days at family level. (6) S6 Fig. LEfSe results displaying the significantly differed bacterial order among the succession days. (7) S7 Fig. Similarity of microbial communities from the biofilm and seawater samples, as illustrated by UPGMA hierarchical clustering and (8) S1 Table. Glimpse of Illumina-generated reads before and after applying quality control criteria and values of alpha diversity indices. (PDF) [file pone.0257961.s001.pdf]

Supplemental Material for the manuscript entitled "Bacterial community structure of early-stage biofilms is dictated by temporal succession rather than substrate types in the southern coastal seawater of India".

T J Sushmitha[a], Meora Rajeev[a], P Sriyutha Murthy[b], S Ganesh[c], Subba Rao Toleti[b], Shunmugiah Karutha Pandian[a, *]

[a] Department of Biotechnology, Alagappa University, Karaikudi, Tamil Nadu, India.

[b] Water and Steam Chemistry Division, Bhabha Atomic Research Centre Facilities, Kalpakkam, Tamil Nadu, India.

[c] Department of Chemistry, Scott Christian College, Nagercoil, Tamil Nadu, India.

S1 Figure

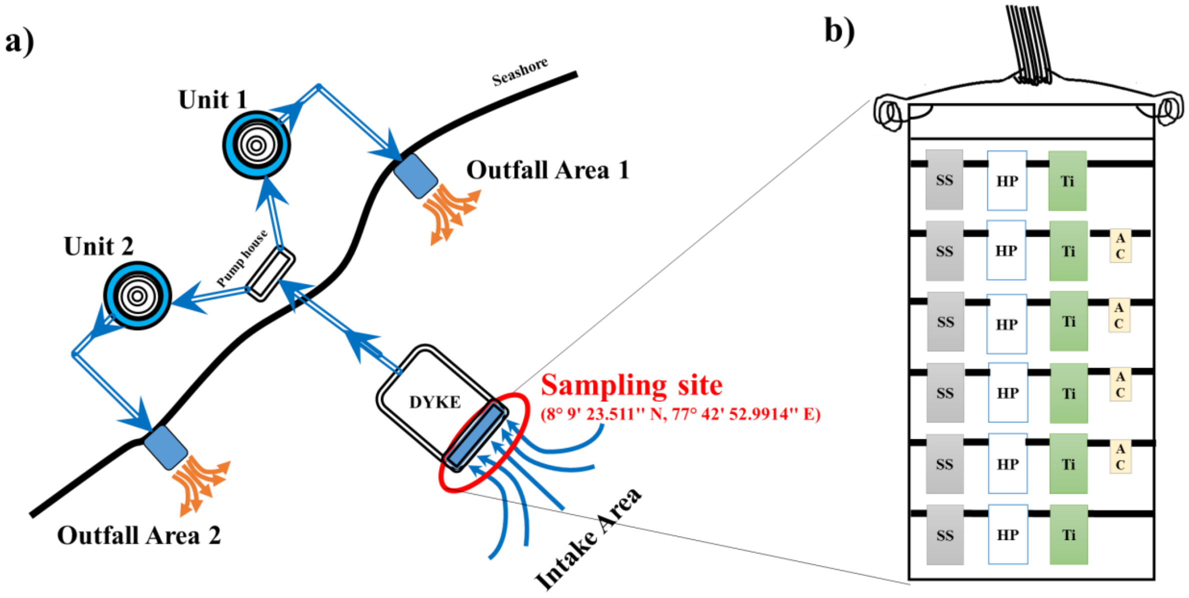

S1 Fig. Setup employed for the development of biofilm in the intake area of a coastal power plant located in the southern coastal region of India. a) Schematic representation of the sampling location showing the study area and location of the immersion sites (intake area of the power plant). b) The polypropylene frames were designed to contain 6 nos. of three artificial substrata, including stainless steel (SS), high-density polyethylene (HP) and titanium (Ti). Additionally acrylic coupons were fixed for the microscopic analysis.

S2 Figure

a)

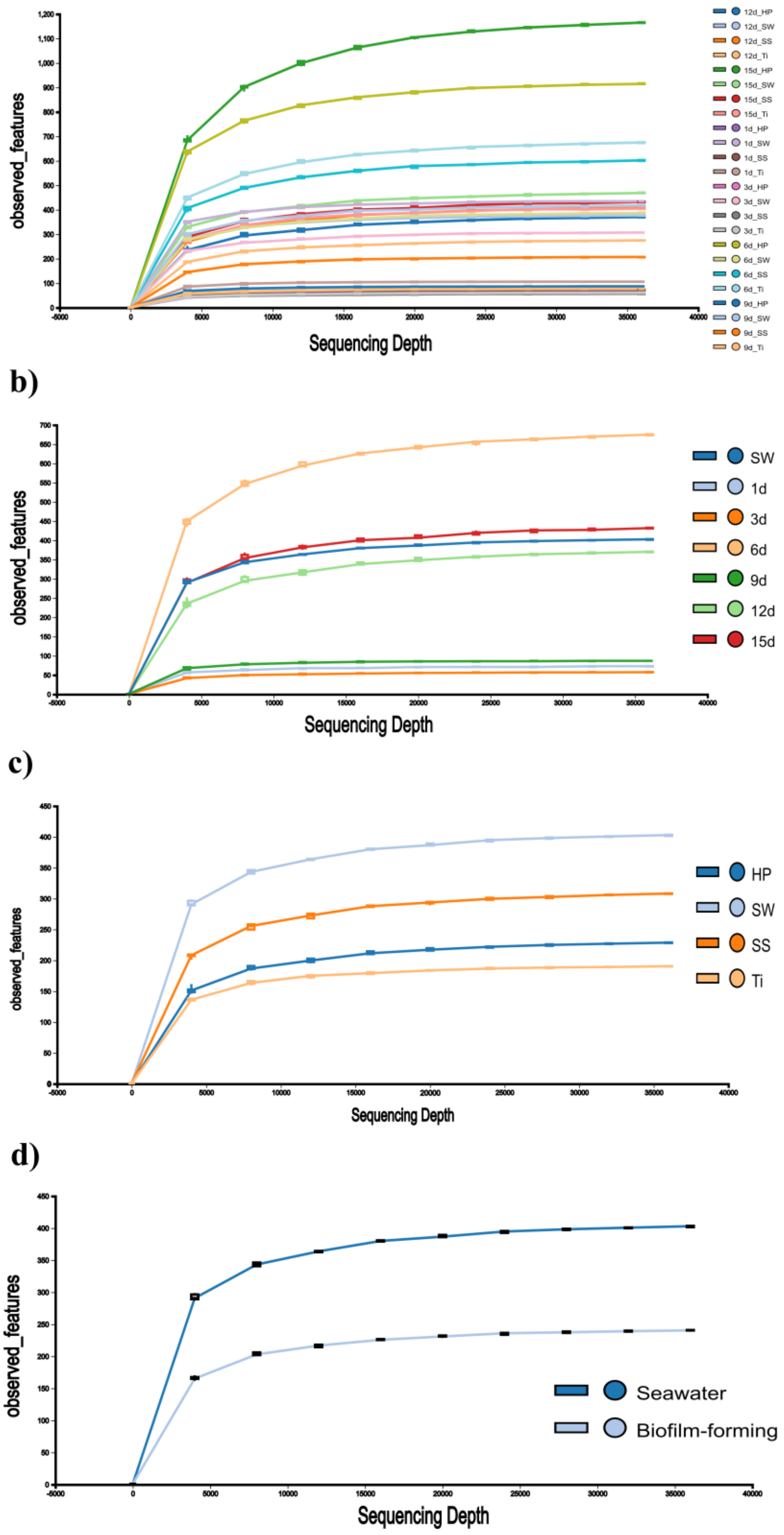

S2 Fig. Rarefaction curves displaying the observed features with an increasing number of reads based on a) samples, b) days, c) substrate and d) between biofilm and seawater communities. Curves reaching asymptote denote adequate sampling depth for the analyzed samples of both biofilm-forming and seawater bacterial communities.

S3 Figure

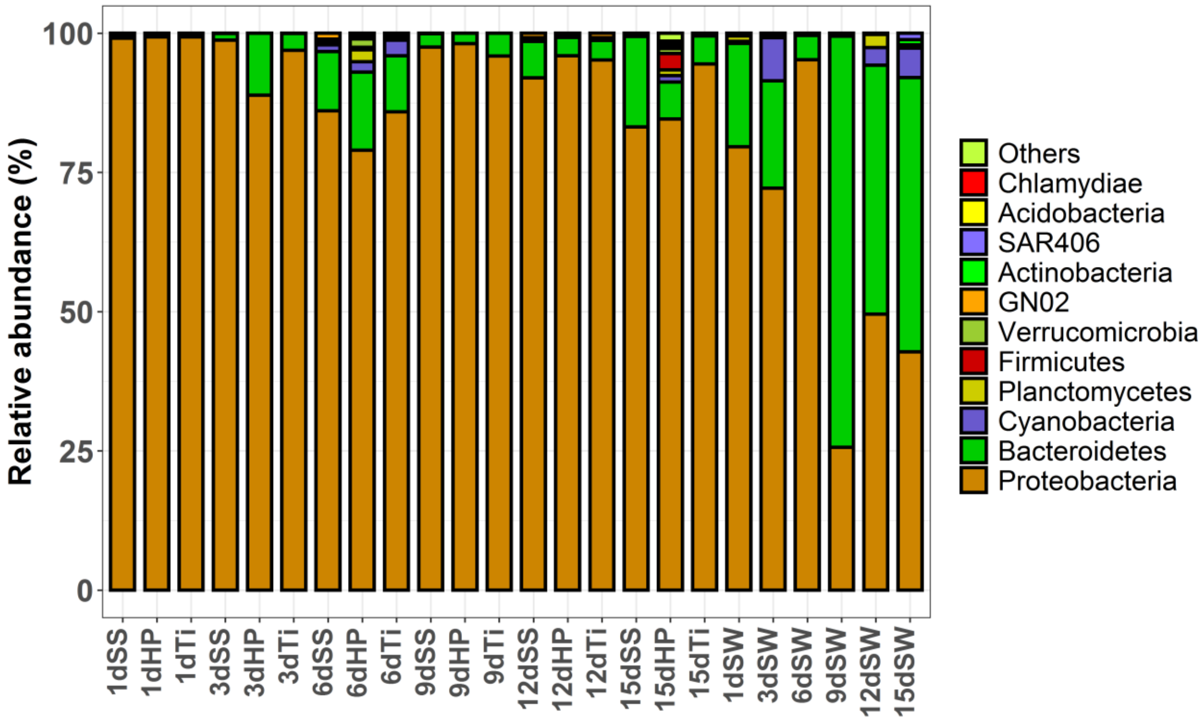

S3 Fig. Taxonomic composition and abundance distribution of biofilm-forming and seawater bacterial communities at the phylum level. Relative abundance of phyla that constitute for more than 0.5% of the total population are only shown in plot.

S4 Figure

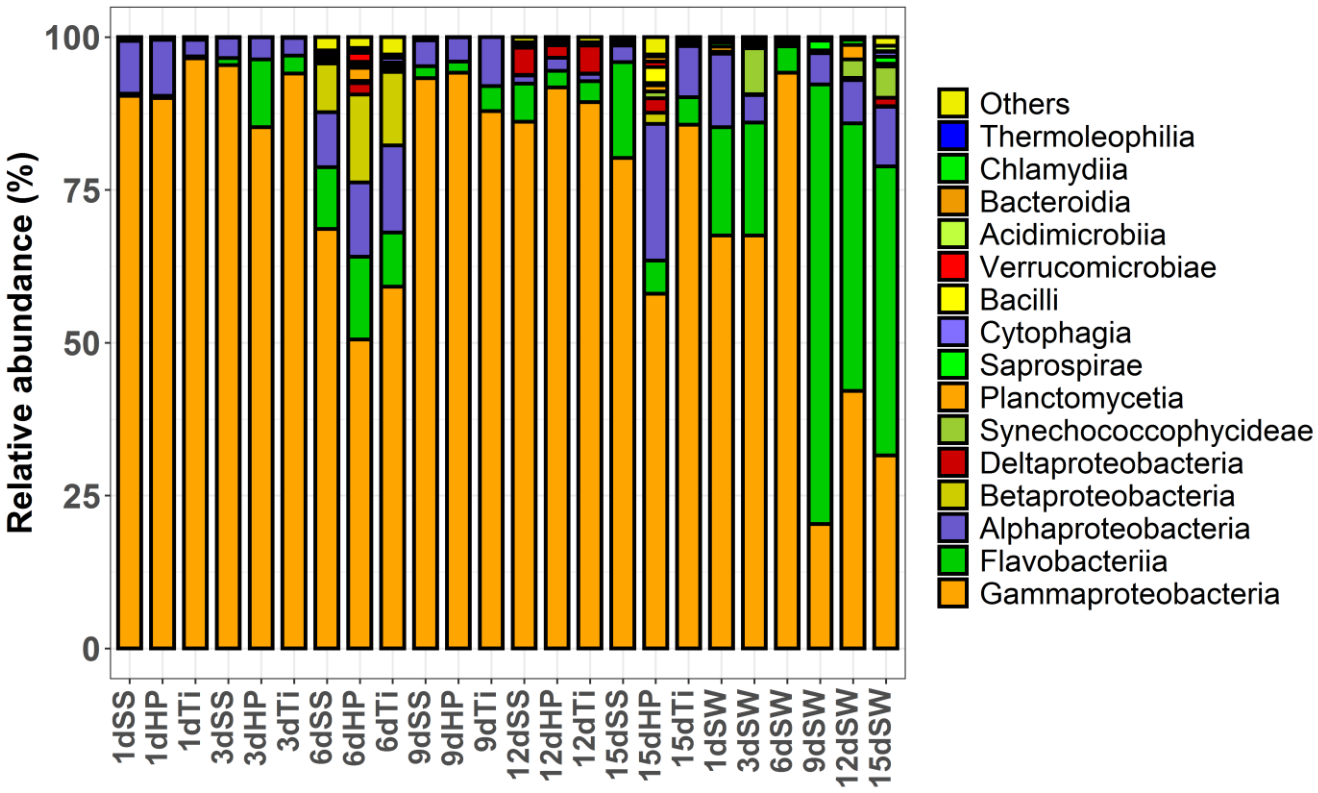

S4 Fig. Taxonomic classification of biofilm-forming and seawater bacterial communities at the class level over different days. Relative abundance of classes that constitute for more than 1% of the total community is only shown in plot.

Figure S5

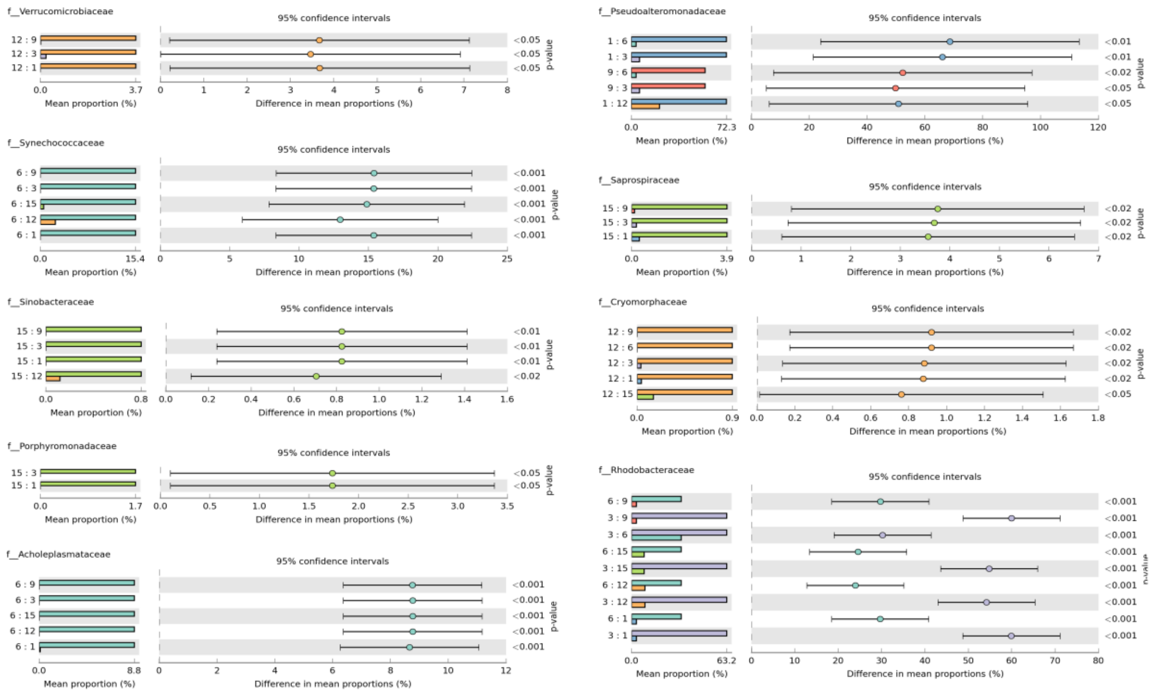

S5 Fig. Bar plots with extended errors displaying the proportional differences of the significantly differed bacterial taxa among the succession days at family level. Significant differences were identified in STAMP software using ANOVA followed by Tukey-Kramer analysis for multiple groups.

S6 Figure

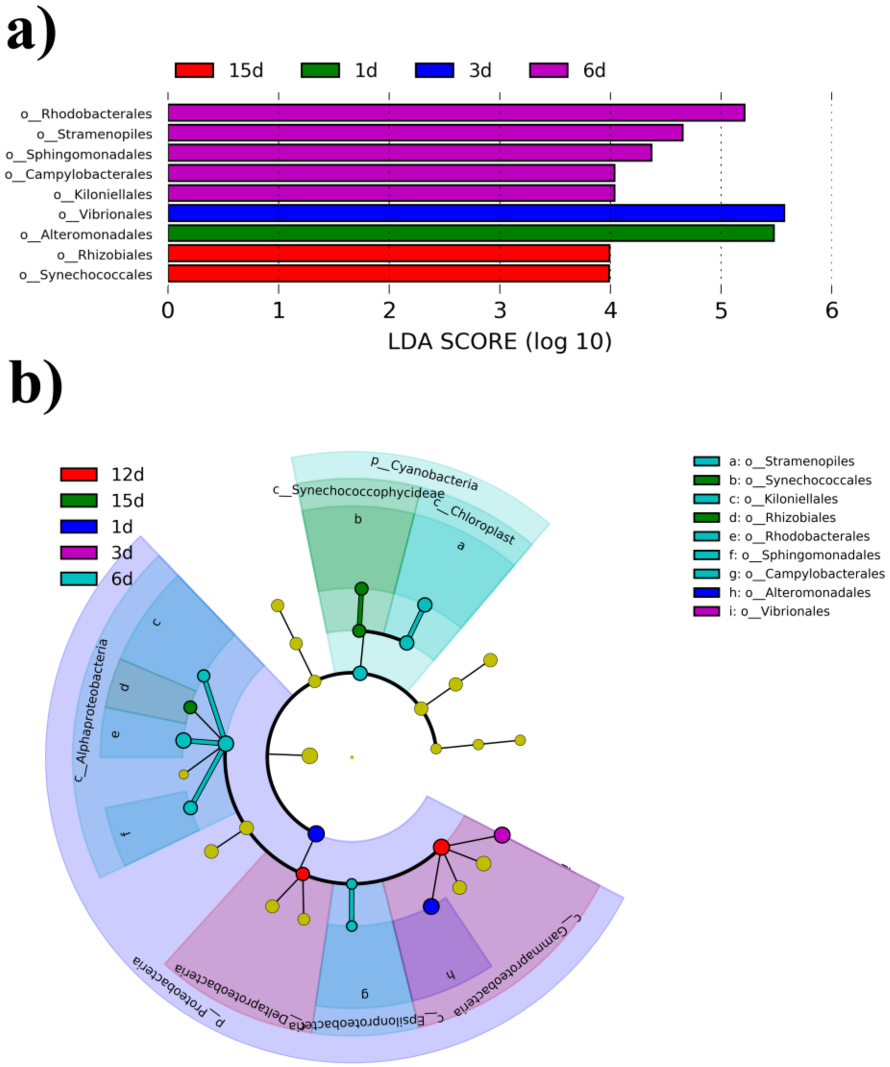

S6 Fig. LEfSe results displaying the significantly differed bacterial order among the succession days. Bacterial orders with LDA scores > 3 are displayed.

S7 Figure

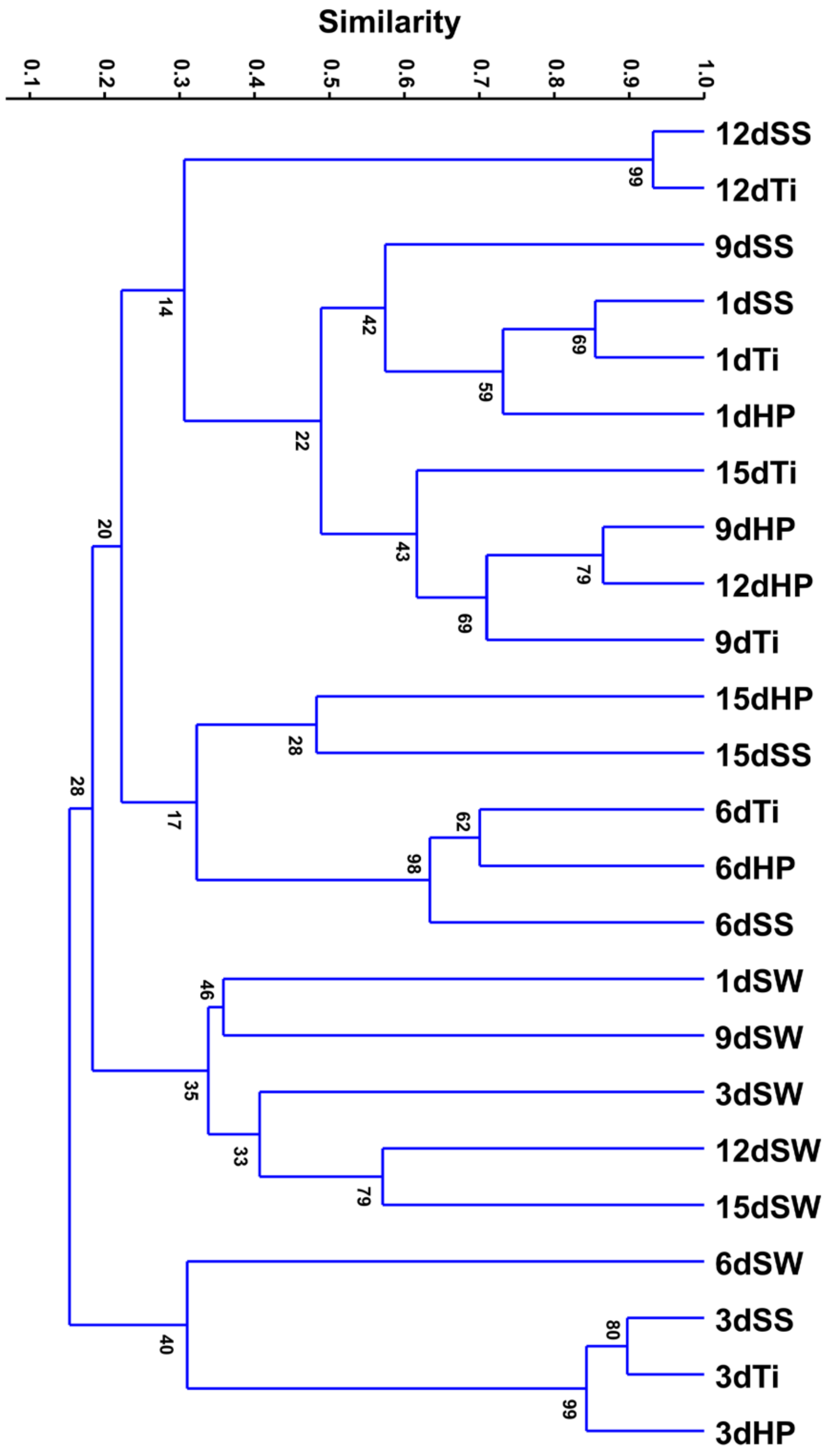

S7 Fig. Similarity of microbial communities from the biofilm and seawater samples, as illustrated by UPGMA hierarchical clustering. Community similarities in the biofilm and seawater samples calculated based on the feature composition and abundance are shown.

S1 Table Glimpse of Illumina-generated reads before and after applying quality control criteria and values of alpha diversity indices.

**Sample ID**	**Days**	**Substrate**	**No. of raw reads (R1+R2)**	**DADA2 quality-filtered reads**	**Reads hit the reference**	**No. of observed OTUs**	**Faith's_PD**	**Evenness**	**Shannon**
**1dSS**	Day 1	SS	199332	65471	44057	73	7.182787	0.432877	2.679431
**1dHP**		HP	190332	64638	37866	70	6.672061	0.462463	2.834569
**1dTi**		Ti	149046	54789	54003	106	9.580465	0.436425	2.936232
**3dSS**	Day 3	SS	206474	77293	59040	59	6.273024	0.594152	3.495182
**3dHP**		HP	208344	77063	58211	56	5.661634	0.61043	3.544985
**3dTi**		Ti	184178	68817	54027	60	7.063592	0.589791	3.48383
**6dSS**	Day 6	SS	206284	60057	50479	599	42.33142	0.731554	6.749621
**6dHP**		HP	177368	48313	45436	914	56.48003	0.826573	8.13021
**6dTi**		Ti	182000	56039	51507	671	44.44475	0.744062	6.986864
**9dSS**	Day 9	SS	175828	56480	44329	206	18.19357	0.483522	3.716592
**9dHP**		HP	176262	61864	43544	87	6.407486	0.545242	3.512966
**9dTi**		Ti	184896	65621	46083	77	7.530526	0.514448	3.223936
**12dSS**	Day 12	SS	207944	69025	56513	411	31.22621	0.535223	4.647342
**12dHP**		HP	217266	72036	52879	371	34.35319	0.550882	4.701927
**12dTi**		Ti	186590	62824	49364	275	23.87572	0.488349	3.95723
**15dSS**	Day 15	SS	186146	61995	52712	429	32.32162	0.597116	5.22168
**15dHP**		HP	199142	67154	65488	1167	82.32774	0.692201	7.052553
**15dTi**		Ti	203400	64307	51382	403	30.04578	0.61617	5.332727
**1dSW**	SW	SW	191112	47846	35998	435	25.13319	0.741129	6.495897
**3dSW**		SW	164180	46739	37630	307	25.19956	0.674401	5.571966
**6dSW**		SW	217476	70683	50881	387	22.87895	0.675333	5.80529
**9dSW**		SW	188668	58931	51130	421	27.46509	0.577012	5.030204
**12dSW**		SW	200360	59314	50563	376	26.97134	0.759574	6.497845
**15dSW**		SW	205866	58723	48527	466	36.60757	0.747899	6.62952
